# On the Microstructure and Isothermal Oxidation of Silica and Alumina Scale Forming Si-23Fe-15Cr-15Ti-1Nb and Si-25Nb-5Al-5Cr-5Ti (at.%) Silicide Alloys

**DOI:** 10.3390/ma12071091

**Published:** 2019-04-02

**Authors:** Ofelia Hernández-Negrete, Panos Tsakiropoulos

**Affiliations:** Department of Materials Science and Engineering, Sir Robert Hadfield Building, The University of Sheffield, Mappin Street, Sheffield S1 3JD, UK; ochernandeznegrete@gmail.com

**Keywords:** coatings, intermetallics, silicides, pest oxidation, high temperature oxidation, Nb-silicide based alloys

## Abstract

An Nb-silicide based alloy will require some kind of coating system. Alumina and/or SiO_2_ forming alloys that are chemically compatible with the substrate could be components of such systems. In this work, the microstructures, and isothermal oxidation at 800 °C and 1200 °C of the alloys (at.%) Si-23Fe-15Cr-15Ti-1Nb (OHC1) and Si-25Nb-5Al-5Cr-5Ti (OHC5) were studied. The cast microstructures consisted of the (TM)_6_Si_5_, FeSi_2_Ti and (Fe,Cr)Si (OHC1), and the (Nb,Ti)(Si,Al)_2_, (Nb,Cr,Ti)_6_Si_5_, (Cr,Ti,Nb)(Si,Al)_2_ (Si)_ss_ and (Al)_ss_ (OHC5) phases. The same compounds were present in OHC1 at 1200 °C and the (Nb,Ti)(Si,Al)_2_ and (Nb,Cr,Ti)_6_Si_5_ in OHC5 at 1400 °C. In OHC1 the (TM)_6_Si_5_ was the primary phase, and the FeSi and FeSi_2_Ti formed a binary eutectic. In OHC5 the (Nb,Ti)(Si,Al)_2_ was the primary phase. At 800 °C both alloys did not pest. The scale of OHC1 was composed of SiO_2_, TiO_2_ and (Cr,Fe)_2_O_3_. The OHC5 formed a very thin and adherent scale composed of Al_2_O_3_, SiO_2_ and (Ti_(1−x−y)_,Cr_x_,Nb_y_)O_2_. The scale on (Cr,Ti,Nb)(Si,Al)_2_ had an outer layer of SiO_2_ and Al_2_O_3_ and an inner layer of Al_2_O_3_. The scale on the (Nb,Cr,Ti)_6_Si_5_ was thin, and consisted of (Ti_(1−x−y)_,Cr_x_,Nb_y_)O_2_ and SiO_2_ and some Al_2_O_3_ near the edges. In (Nb,Ti)(Si,Al)_2_ the critical Al concentration for the formation of Al_2_O_3_ scale was 3 at.%. For Al < 3 at.% there was internal oxidation. At 1200 °C the scale of OHC1 was composed of a SiO_2_ inner layer and outer layers of Cr_2_O_3_ and TiO_2_, and there was internal oxidation. It is most likely that a eutectic reaction had occurred in the scale. The scale of OHC5 was α-Al_2_O_3_. Both alloys exhibited good correlations with alumina forming Nb-Ti-Si-Al-Hf alloys and with non-pesting and oxidation resistant B containing Nb-silicide based alloys in maps of the parameters δ, Δχ and VEC.

## 1. Introduction

The search for structural materials with improved ultra-high temperature capabilities beyond those of Ni-based superalloys has concentrated on refractory metal intermetallic composites (RMICs), among which Nb-silicide based alloys (also known as Nb silicide in situ composites) continue to attract much attention because of their desirable densities, high liquidus temperatures and their offering of a balance of properties. These new alloys, like the Ni-based superalloys, will require a coating system to reduce the temperature of the metal surface (substrate) and enhance resistance to oxidation in the environments where they will operate.

A coating system on Nb-silicide based alloys could be of the thermal barrier type consisting of bond coat (BC), thermally grown oxide (TGO) and top coat (TC). In a materials system consisting of the substrate, BC, TGO, TC and the environment, a systems approach is needed to establish design methodologies (approaches) and to control and improve the performance. A multi-material BC could be used, where the BC components should enable the adherence of other components of the coating system and protect the substrate from interstitial contamination. The BC could include a silicide coating alloy and other components, for example a diffusion barrier consisting of a Laves phase containing layer and/or a platinum group metal layer [1] and/or alumina forming alloy(s) [2,3]. The temperature-time history of the TGO could be the dominant factor governing the life of the coating system. The selection of silicide coating alloys for Nb-silicide based alloys could benefit from earlier research on coatings for Nb alloys.

Nb alloys (not Nb-silicide based alloys) have been considered for advanced aerospace vehicles, flight propulsion systems and advanced gas turbines owing to the high melting point of Nb, their strength potential to 1650 °C and their density. Because of the severe degradation of Nb alloys at elevated temperatures, coatings were developed to provide resistance to oxidation, thermal fatigue, hot gas erosion, particle abrasion, impact damage and strain induced cracking [4]. Techniques used to form protective coatings utilised vapour transport and diffusion (e.g., pack cementation) and liquid-solid diffusion (e.g., fused slurry, hot dipping). Chemical vapour deposition, plasma spraying techniques and cladding were also used [4].

The Nb alloy development research confirmed the effectiveness of Ti and Al in improving the oxidation resistance of Nb. These elements also enhance the oxidation resistance of Nb-silicide based alloys [5]. The coating development research demonstrated that plasma sprayed Si-Mo-Al-Cr-B (LM-5) coating with a Nb-Ti-Cr-Al-Ni intermediate layer provided excellent oxidation protection to Nb alloys at 1150 °C (for about 1000 h) and 1480 °C (≈ 100 h). Aluminide coatings on Nb alloys offered oxidation protection for shorter times and at lower temperatures compared with silicide coatings, and also were susceptible to pest oxidation [4].

The oxidation of alloyed MoSi_2_ at intermediate and high temperatures depends on alloying additions [6,7]. Silicide coatings based on the Mo disilicide were considered for Nb alloys [4]. Silicide coatings (not just MoSi_2_ based) on Nb alloys could provide oxidation protection below 1370 °C. Pack cementation Si-Cr-Al silicide based coating alloys with the addition of Ti gave duplex coating systems with excellent oxidation resistance up to 1425 °C, no pesting and no rapid oxidation of the substrate at the base of cracks of the silicide coating. The low ductility of silicide coatings was addressed with coating systems that included ductile layer(s) to absorb strain induced by impact, deformation or thermal stress. Ductile layers based on Fe-Cr-Al alloys were considered but their use was not pursued owing to their catastrophic oxidation in the range 1315 °C to 1370 °C. Instead Nb-Cr-Ti layers were used [4].

The compositions near the top surface and mid-thickness of an Al free silicide coating about 60 μm thick on a Nb-10W-1Zr-0.1C (wt.%) alloy (D-43) respectively were 65Si-19Cr-15Ti-1Nb and 62Si-18Cr-10Ti-10Nb (at.%). Complex silicide alloys were the most effective coatings for Nb alloys, they did not suffer from pest oxidation and could offer oxidation protection for over 1000 h at 1200 °C. Examples are the Si-20Cr-20Ti (R-512A) and Si-20Cr-20Fe (R-512E) coatings (wt.%) that were developed by Sylvania Electric Products, Inc., Hicksville, N.Y (often referred to as Sylvania coatings and also known as HITEMCO coatings). In oxidation-erosion, silicides of the Si-Cr-Ti type proved most protective, while in thermal fatigue R-512E type silicides were superior [4].

Dense, continuous and adherent Al_2_O_3_ or SiO_2_ oxides protect alloys from oxidation at high temperatures (T > 1000 °C). These oxides are the most protective because of their high thermodynamic stability and the low diffusivities for anions and cations. An understanding of the microstructures that govern the performance of materials used in coatings is needed to provide a sound basis for developing advanced coating systems for a particular family of substrates. 

An alumina or silica forming BC alloy applied on a substrate could consist of three parts after oxidation. The oxide on the top (primary barrier), the coating alloy which acts as a reservoir for the oxide formation and a diffusion zone [8]. Diffusion barrier(s) can minimise interdiffusion between coating and substrate. The time to failure of the materials system (substrate + BC) would depend on how the three parts perform as oxygen barriers and its mechanical performance would be affected (i) by reservoir ductility, (ii) coating/substrate interdiffusion and (iii) craze cracks in the reservoir because of coefficient of thermal expansion (CTE) mismatch. Further, (ii) and (iii) can also have a significant effect on the oxidation performance [9].

The metallurgy of refractory metal alloys also has been interested in combining either silicide or other reservoirs and controlled composition silica glasses (primary barrier). The composition of the glass formed on a silicide coating is a function of the composition of the reservoir, temperature and pressure, and varies within these three parameters. Research on coatings for Nb-silicide based alloys has exploited silicide based coatings (e.g., [8,10,11]). The knowledge gained from earlier research on coatings for refractory metal alloys (not RMICs) and from recent research on Nb-silicide based alloys was used in the research presented in this paper.

Type, number and distribution of defects and interdiffusion profiles are interrelated respectively with the coating process and the substrate used. In the absence of defects, coating life is limited by diffusional processes at low and high temperatures, and by evaporation or melting at ultra-high temperatures. At temperatures above approximately 1650 °C, the evaporation rate of SiO_2_ becomes significant, as do the vapour pressures of SiO in equilibrium with Si and SiO_2_ and of Si in equilibrium with the Si rich (i.e., the higher) silicides. With alumina forming coatings the vapour pressure of AI is appreciable at higher temperatures. 

The aims of the research presented in this paper were (a) to provide an insight into the design and selection of metallic materials for a coating system for Nb-silicide based alloys, (b) to highlight issues that require new levels of understanding and (c) to find out if alloys based on Si-Cr-Ti-X (X = Al, Fe, Nb) systems are worthy of further research to ascertain their application as BC alloys for Nb-silicide based alloys. Two alloys have been chosen for consideration in this paper. The objectives of the research were (i) to study the microstructures of the cast and heat treated alloys, (ii) to evaluate their isothermal oxidation at 800 and 1200 °C and (iii) to “define” pathway(s) for alumina and/or silica forming BC alloys for Nb-silicide based alloys in maps of the parameters VEC, δ and Δχ.

In this paper, we report for the first time our research on two new alloys of the aforementioned system. The alloys were not studied as coatings applied on a Nb-silicide based substrate in order to eliminate the effects of substrate and coating process on microstructures and isothermal oxidation [2,3]. 

The structure of the paper is as follows. First, we discuss how the alloy compositions were selected. This section is followed by a brief description of the experimental techniques that were used. The results for the cast and heat-treated microstructures of the alloys, and their isothermal oxidation at 800 °C and 1200 °C are then presented. In the discussion, we deliberate on the microstructures of the alloys before we consider their oxidation. The alloys are then compared with the alumina forming Nb-Ti-Si-Al-Hf alloys and Nb-silicide based alloys. Suggestions for future work are given before the summary and conclusions.

## 2. Design of Alloys

Our goal was to design and develop SiO_2_ and/or αAl_2_O_3_ scale forming silicide alloys. These alloys should not pest. The design of the alloys benefited from the research reported in [2,3], the design methodology NICE [5] and knowledge about the oxidation of silicides (see below). The alloys were designed to have (i) no stable Nb solid solution in their microstructures and (ii) Si rich and/or Al and Si rich transition metal silicides.

### 2.1. Why Silicide Coating Alloys?

The research on refractory metals and their alloys has shown that effective primary barriers for use below 1370 °C are silica and silica glasses. Silicide coatings on Nb alloys (not Nb-silicide based alloys) have withstood multiple thousands of cycles from room temperature to 1200 °C to 1370 °C without reservoir spallation [4]. Strains due to the differential expansion between oxide (primary barrier), reservoir or substrate, oxide growth stresses and cracking of the oxide due to differences in temperature could lead to the rapid consumption of the reservoir in the case of alumina [12]. The fact that the elastic moduli of alumina (215 to 413 GPa) and silica (66 to 75 GPa) [13] are different is important. The low elastic modulus of silica minimises the effects of strains. Silica can be vitreous and readily vitrified below 1370 °C by minor additions that can also lower the softening temperature of the glass to about 650 °C, almost completely eliminating strain in the primary oxide as a cause of coating failure. Cristobalite and tridymite form as the scales devitrify. The growth rate of the scale and the activation energy for diffusion through SiO_2_ depend on whether the scale is crystalline or amorphous [14].

Silicide coatings can be modified with B or Ge. Both elements are important additions in Nb-silicide based alloys [15,16] because they contribute to the suppression of pest oxidation and also improve high temperature oxidation resistance [17]. B_2_O_3_ or GeO_2_ solute in SiO_2_ results in a glass with higher fluidity at a lower temperature to heal cracks. The CTE values of B_2_O_3_-SiO_2_ and GeO_2_-SiO_2_ are significantly higher than pure SiO_2_ [18].

The low softening temperature is advantageous because there might be a marked expansion mismatch between silicide coating and Nb-silicide based alloy substrate. Craze cracks would develop in the reservoir during cyclic oxidation. During every thermal cycle, oxide would build up in these craze cracks. Unless the oxide can be extruded on heat-up, shear failure would eventually occur between the reservoir and the substrate. Alumina, which does not soften until above 1650 °C, cannot be extruded from craze cracks. This limits the cycle performance of aluminide reservoirs.

### 2.2. Which Silicide(s)?

The MSi_2_ has the highest Si activity. When MSi_2_ oxidises the lower MSi or M_5_Si_3_ silicides can form (M is transition metal). The CrSi_2_ and NbSi_2_ have the same crystal structure (C40 compounds) [19]. The structures of these disilicides and the C54-TiSi_2_ are closely related and can be regarded as alternative stackings of layers that are topologically similar to bcc (110) planes [20]. The nearest neighbour environments in the C40 and C54 structures are fully equivalent. The face centred orthorhombic C54 structure of TiSi_2_ is the stable one, but there exists a base centred orthorhombic C49 structure that is metastable. The C49 → C54 transformation temperature of TiSi_2_ decreases with alloying additions and the decrease depends on the electronegativity of the ternary addition. Nb decreases this transformation temperature by 50 °C [21]. The FeSi and CrSi have the same crystal structure (B20 compounds) [19].

At 800 °C the NbSi_2_ fails catastrophically but the disilicides of Cr and Ti, and the (Ti,Cr)Si_2_ and (Ti,Cr,Nb)Si_2_ have excellent oxidation resistance. The FeSi_2_ forms SiO_2_ in the range 500 °C to 1000 °C [22,23] but has been reported to be susceptible to pest oxidation [24]. The FeSi also forms SiO_2_ [25]. At temperatures above 1000 °C, the CrSi_2_ forms Si containing scales that tend to be non-adherent. As temperature increases there is a volatilisation of CrO_3_. At 1315 °C the NbSi_2_ has very poor oxidation resistance compared with the outstanding resistance of TiSi_2_ and the CrSi_2_ losses weight and the scale spalls off on thermal cycling. The (Ti,Cr)Si_2_ gains weight rapidly but the addition of Nb in (Ti,Cr,Nb)Si_2_ greatly slows down the oxidation. [12]. The literature points to the fact that tolerance for Nb by disilicides is extremely important in coatings formed by diffusion into Nb alloys.

The oxidation resistance of Nb_5_Si_3_ at 800 °C and 1315 °C is poor. This silicide exhibits solubility for transition metals, simple metals and metalloid elements [26] but is likely to suffer from environmental embrittlement [27]. At 800 °C the oxidation of Cr_5_Si_3_ is better than that of Ti_5_Si_3_. The (Ti,Cr)_5_Si_3_ has good oxidation resistance at 800 °C and 1315 °C where its weight gain is small. At both temperatures the (Ti,Cr)_5_Si_3_ and (Ti,Cr,Nb)_5_Si_3_ have equal or superior oxidation resistance compared with the disilicides containing these elements. A tolerance for Nb is particularly lacking in silicides that do not contain Cr and Ti. The significance of having comparable oxidation performances for both M_5_Si_3_ and MSi_2_ is quite marked. According to Metcalfe and Stetson, “two mils of NbSi_2_ would convert to four mils of Nb_5_Si_3_ at 1315 °C in about 51 h”. Only Cr-Si-Ti compositions forming (Ti,Cr,Nb)_5_Si_3_ would be expected to have reliable oxidation performance beyond the time necessary for conversion of all the MSi_2_ to M_5_Si_3_ [12]. The Fe_5_Si_3_ forms Fe_2_O_3_ with linear oxidation kinetics, and the sulfidation resistance of the scale is better than that of Cr_2_O_3_ or Al_2_O_3_ scales.

Nb behaves well in sulfidation conditions [28]. Combining an oxidation resistant element like Cr with a sulfidation resistant element like Nb has led to Cr-Nb alloys (e.g., Cr-40Nb (wt.%)) with oxidation and sulfidation resistance [29]. The addition of Al in Nb at concentrations exceeding 5 at.% also increased sulfidation resistance compared with pure Nb [30]. Resistance to sulfidation was also exhibited by Nb-38Al-4Si and Nb-35Al-6Si (at.%) alloys [31].

### 2.3. Selection of Alloys

The aforementioned literature guided us to consider coatings containing the elements Al, Cr, Fe, Nb, Si, Ti. We were interested in Fe because of the earlier research on Sylvania type coatings (see introduction) and because Fe as an addition with Cr in Nb-silicide based alloys [32] promotes the formation of C14 Laves phase [33] that enhances their oxidation resistance. The design of the αAl_2_O_3_ forming Nb-Ti-Si-Al-Hf alloys [2,3] was guided by the design methodology NICE [5] and showed that three key parameters based on electronegativity (Δχ), atomic size (δ) and the number of valence electrons per atom filled into the valence band (VEC) described well their alloying behavior. We wanted to explore other areas in the maps of the parameters Δχ,δ and VEC that were published in Figure 11 in [3] with silicide coating alloys with microstructures with MSi_2_, M_5_Si_3_ and M_6_Si_5_ type silicides, the latter because it can be in equilibrium with the former two [34,35]. Keeping in mind the requirements (i) and (ii) (see the start of this section) the compositions of Sylvania silicide coating alloys (see introduction), the oxidation of silicides (see previous two sections), the alloying elements of interest and available phase equilibria data for the Cr-Si-Ti [34], Fe-Si-Ti [36], Cr-Fe-Si [37] and Cr-Nb-Si [35] systems, we selected the Si concentrations of about 45 at.% and 60 at.%, and designed alloys to be located (1) in the right hand side in the Δχ versus VEC map of alumina forming Nb-Ti-Si-Al-Hf alloys [3] with (a) Δχ in-between the values corresponding to Zone A of the alloy MG7 (Nb_1.3_Si_2.4_Ti_2.4_Al_3.5_Hf_0.4_) [2] and the alloys MG5 (Nb_1.45_Si_2.7_Ti_2.25_Al_3.25_Hf_0.35_), MG6 (Nb_1.35_Si_2.3_Ti_2.3_Al_3.7_Hf_0.35_) and MG7 [3] and inside the “forbidden” range of Δχ values of the Nb solid solution [38], (b) VEC higher than the values of the alloys MG5, MG6 and MG7 [3] and (c) δ lower than the alloys MG5, MG6 and MG7 but higher than the Zone A of the alloy MG7 [2], and (2) in the top part in the Δχ versus VEC map of alumina forming Nb-Ti-Si-Al-Hf alloys [3] with (d) Δχ “similar” to the bulk and top of the alloys MG5, MG6 and MG7 [2,3] and inside the “forbidden” range of Δχ values of the Nb solid solution [38] and (e) VEC and δ higher than the alloys MG5, MG6 and MG7. We designed a number of coating alloys. In this paper we report on two of these alloys, the nominal compositions (at.%) of which respectively were 46Si-23Fe-15Cr-15Ti-1Nb (OHC1) and 60Si-25Nb-5Al-5Cr-5Ti (OHC5).

## 3. Experimental

Small buttons of the alloys were prepared from pure elements (≥99.9 wt.% purity) in a Ti gettered Ar atmosphere using arc melting with a water cooled copper crucible, a non-consumable tungsten electrode, a voltage of 50 V and a current of 650 A. Each alloy was melted five times to homogenize its composition. The heat treatments were carried out in an alumina tube furnace in a Ti gettered Ar atmosphere. The alloys were wrapped in Ta foil to minimize contamination by oxygen and were placed in an alumina crucible. The heat treated alloys were furnace cooled.

Conventional metallographic preparation of specimens was used. This involved mounting in bakelite, grinding with SiC paper (from 120–1200 grit) and then to grade 4000 and cashmere cloth polishing with 1μm diamond suspension. The microstructures were characterised using scanning electron microscopy (SEM) and X-ray diffraction (XRD). A Philips PSEM 500 SEM (SEM, Philips-ThermoFisher Scientific, Hillsboro, OR, USA), Jeol JSM 6400 and Inspect F FEG SEM (SEM, Jeol, Tokyo, Japan) were used. The back scatter electron (BSE) mode was mainly used to study the microstructures with qualitative and quantitative energy dispersive X-ray spectroscopy analysis of the alloys and phases. EDS standardization was performed using specimens of high purity Nb, Ti, Cr, Fe, Si, Al, and Co standards that were polished to 1 μm finish. The EDS was calibrated prior to analysis with the Co standard. At least five large area analyses were performed in the top, bulk and bottom of the button and at least ten analyses were obtained from each phase (spot analyses) with size ≥5 μm to determine actual compositions. 

A Siemens D500 XRD diffractometer (XRD, Hiltonbrooks Ltd, Crew, UK) with CuKα radiation (λ = 1.540562 Å), 2θ from 20°–120° and a step size of 0.02° was used. For glancing angle XRD (GXRD) a Siemens D5000 diffractometer (Hiltonbrooks Ltd, Crew, UK) with Cu Kα1 and Kα2 radiation (λ = 1.54178 Å), 2θ from 10°–100° and a step size of 0.02° was used. Peaks in the XRD diffractograms were identified by correlating data from the experiments with that from the JCPDS data (International Centre for Diffraction Data). The scan type used for GXRD was detector scan while for regular specimens it was locked coupled. Prior to GXRD experiments the glancing angle was selected with the aid of the AbsorbDX software which evaluates the X-ray penetration depth for particular glancing angle conditions.

The isothermal oxidation of the alloys was studied at 800 °C and 1200 °C for 100 h using a Netzsch STA F3 TG/DSC analyser (Netzch Gmbh, Waldkraiburg, Germany) with a SiC furnace with air flow rate of 20 mL/min and with heating and cooling rates of 3 °C/min. Cubic specimens of size 3 mm × 3 mm × 3 mm and polished to 800 grit SiC finish were used for the thermo-gravimetry (TGA, SEM) experiments. For the DSC experiments a Rh/Pt furnace was used in the Netzsch STA F3 TG/DSC analyser with an Ar flow rate of 20 mL/min. The specimens for thermal analysis were selected from the bulk of the cast buttons.

## 4. Results

### 4.1. Cast Alloys

**OHC1**: The actual composition (at.%) of the cast alloy (OHC1-AC) was Si-23Fe-14.5Cr-15Ti-1Nb, close to the nominal one. This composition was the average of the analyses taken from the top, center and bottom of the button. There was macrosegregation of Ti, Cr and Fe in the button. The concentrations of Ti, Cr and Fe were respectively in the range 8.7–15.7 at.%, 8.1–15.6 at.% and 22–35.5 at.%, with the bottom of the button rich in Fe and lean in Ti and Cr. The parts of the button in direct contact with the water cooled copper crucible (“chill zones”) were richer in Fe (average value 34.0 at.%) and leaner in Cr, Ti and Nb with average values of 8.9, 9.6 and 0.4 at.%, respectively. A “layered” microstructure like the one reported for the alloy MG7 in [2] was not observed.

**Figure 1:**
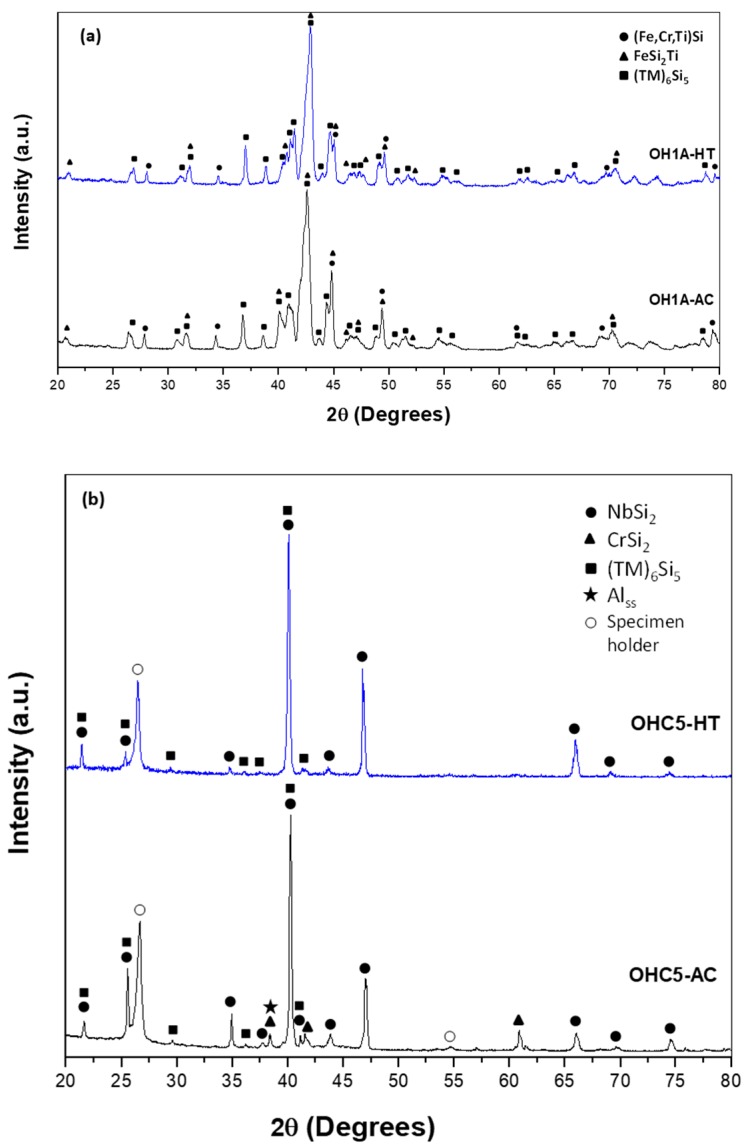
X-ray diffractograms of the alloys (**a**) OHC1 and (**b**) OHC5 in the cast and heat-treated conditions.

The XRD (Figure 1a) and EDS data confirmed that the microstructure of the alloy consisted of the (TM)_6_Si_5_, FeSi_2_Ti and (Fe,Cr,Ti)Si compounds, where TM = Nb, Ti, Fe, Cr. The (TM)_6_Si_5_ compound is also known as the T phase in the Ti-Cr-Si system [34]. It crystallizes in the orthorhombic system with the V_6_Si_5_ as its prototype and has space group Ibam [34]. Its average composition was 45.9(0.3)Si-23.5(4.3)Fe-13.8(2.7)Cr-15.9(1.4)Ti-0.8(0.3)Nb, where in parenthesis is given the standard deviation. The FeSi_2_Ti compound is the τ_1_ phase in the Fe-Si-Ti system with MnSi_2_Ti prototype. It is orthorhombic with the Pbam space group. Its composition (47(0.5)Si-31.2(1)Fe-7(1.3)Cr-14.1(1.1)Ti-0.2Nb) matched with the composition of the τ_1_ phase reported by Weitzer et al. [39], particularly when the data for the heat treated alloy (OHC1-HT) was taken into account (see below). The FeSi phase with the B20 structure crystallizes in the cubic system with the P2_1_3 space group [19]. The composition of the (Fe,Cr,Ti)Si phase was 51.4(0.2)Si-41.7(0.6)Fe-5.9(0.6)Cr-0.9Ti-0.1Nb.

**Figure 2:**
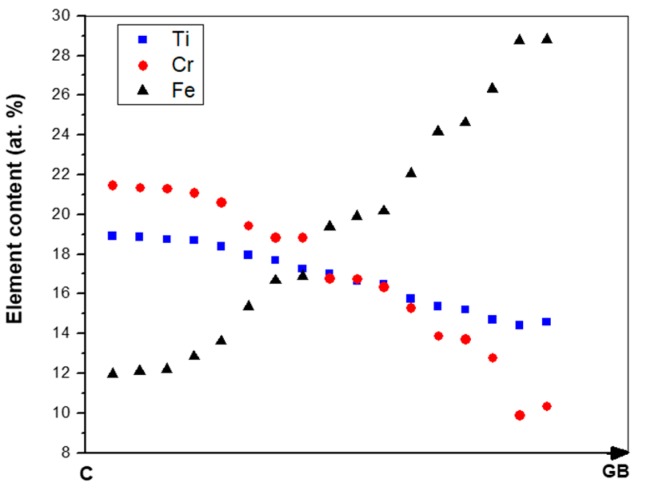
Average Fe, Cr and Ti concentrations from the center (C) of a (TM)_6_Si_5_ dendrite towards its edge (GB).

The microstructure in the top and bulk of the button was the same. The facetted dendrites of (TM)_6_Si_5_ were severely cracked and the transition metal (TM) content varied with location. Figure 2 shows the EDS data for Cr, Fe and Ti for one such dendrite.

**Figure 3:**
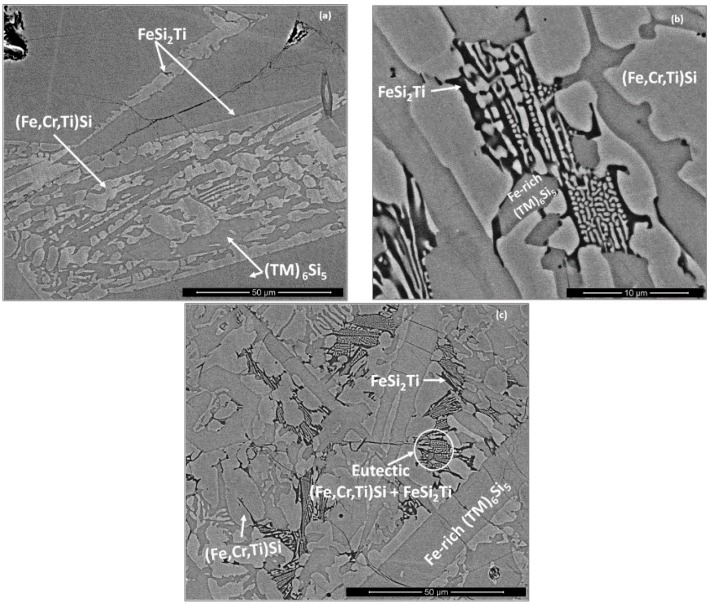
BSE images of the microstructure in (**a**) bulk, ×2000 (**b**) bottom, ×8000 and (**c**) the “chill zone” (×2000) of OHC1-AC.

The (Fe,Cr,Ti)Si compound exhibited an elongated facetted morphology and surrounded a thin dark grey layer of the FeSi_2_Ti phase (Figure 3a). In the lower part of the bulk towards the bottom of the button a very fine lamellar eutectic was observed (Figure 3b,c). This eutectic (50.2(0.2)Si-35.6(0.4)Fe-3.7(0.2)Cr-10.4(0.4)Ti-0.1Nb) consisted of the (Fe,Cr,Ti)Si (bright contrast) and FeSi_2_Ti (dark contrast) phases, the average compositions of which respectively were 50.8(0.4)Si-36.9(1.5)Fe-4(0.5)Cr-8.2(2)Ti-0.1Nb and 50(0.2)Si-31(1.1)Fe-3.4(0.3)Cr-15.3(1.3)Ti-0.2Nb. In the “chill zone” a higher volume fraction of the (Fe,Cr,Ti)Si and FeSi_2_Ti phases was observed. The aforementioned eutectic was also present. In contrast to the results obtained for the (TM)_6_Si_5_ silicide that was observed in the bulk of the alloy, the microsegregation in the (TM)_6_Si_5_ phase was not detected in the “chill zone” and instead only Fe-rich (TM)_6_Si_5_ was observed.

**Figure 4:**
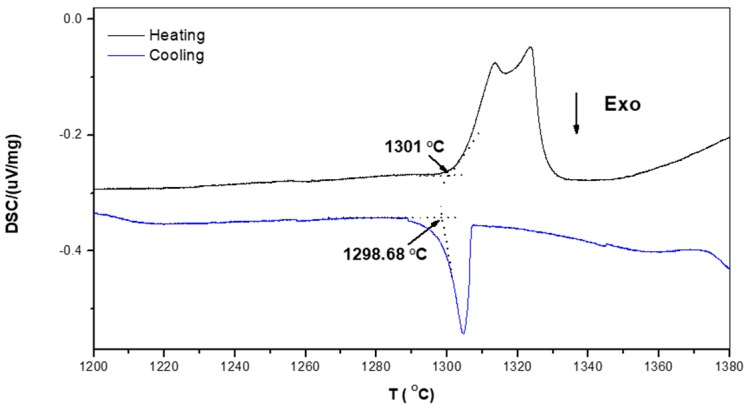
DSC trace of the alloy OHC1.

According to the Fe-Si binary phase diagram [19], the melting point of the FeSi phase is 1410 °C. The Cr and Ti additions will increase the melting temperature of (Fe,Cr,Ti)Si. According to Du and Shuster [40] and Weitzer et al. [39], the melting points of the (TM)_6_Si_5_ and FeSi_2_Ti phases should be above 1500 °C. The DSC trace of the alloy OHC1 (Figure 4) showed a thermal event on heating at about 1300 °C that consisted of a double peak. On cooling there was a single peak at about 1298 °C that could correspond to the crystallization of the previous. The endothermic signal showed a double peak that could be due to heterogeneities in the participating phases. The peaks could correspond to the eutectic FeSi + FeSi_2_Ti observed in the bottom of the alloy and the eutectic reported by Weitzer et al. [39] at 1328 °C (L → FeSi + FeSi_2_Ti). However, they also assigned a peak at 1298 °C to the invariant reaction L + FeSi_2_Ti → FeSi + τ_4_ where τ_4_ = Fe_28.1_Ti_26.3_Si_45.6_. The τ_4_ compound was not observed in the alloy OHC1.

**OHC5**: The actual composition (at.%) of the as cast alloy (OHC5-AC) was Si-25.6Nb-4.6Cr-5.2Ti-5.1Al, very close to the nominal composition. This was the average of the analyses taken from all parts of the button. There was macrosegregation of all the elements. The highest Nb and Si and lowest Al, Cr and Ti concentrations were observed in the bulk of the button. The concentrations of Al, Cr, Nb, Si and Ti respectively were in the range 2.4–6.9, 1.8–6.4, 23.2–29.9, 57.1–63.4 and 2.6–7.6 at.%. 

**Figure 5:**
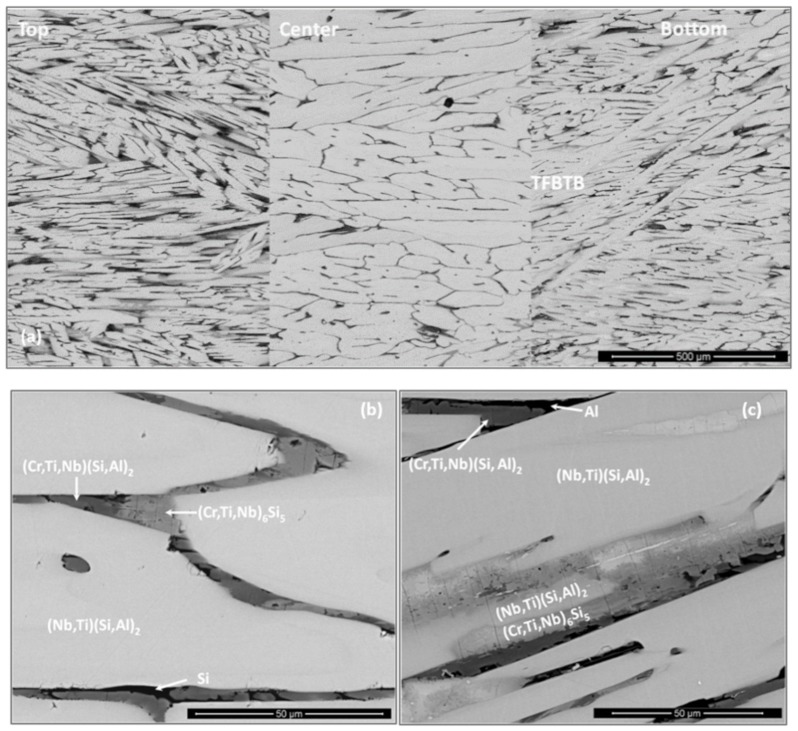
BSE images of the microstructure of the alloy OHC5-AC, (**a**) cross section, ×200 (**b**) bulk, ×2500 and (**c**) bottom (×2000) of the button.

According to the XRD data (Figure 1b) the microstructure of OHC5-AC contained the hexagonal C40 phases NbSi_2_ (JCPDS card 8-450) and CrSi_2_ (JCPDS card 01-079-3529), the orthorhombic (Cr,Ti,Nb)_6_Si_5_ phase (JCPDS card 89-4813), and possibly Al (JCPDS card 4-787). The XRD data and the EDS analyses confirmed the following phases (Nb,Ti)(Si,Al)_2_, (Cr,Ti,Nb)_6_Si_5_, (Cr,Ti,Nb)(Si,Al)_2_, and Si and Al solid solutions (Figure 5b,c) with compositions, respectively 64.2(0.5)Si-31.6(0.6)Nb-2(0.5)Ti-2(0.6)Al-0.4Cr, 45.3(0.4)Si-14.6(5.7)Nb-18(1.2)Ti-20.8(4.8)Cr-1.2(0.5)Al, 58.6(1.6)Si-3.8(1)Nb-10.4(1)Ti-19.3(1.5)Cr-8(1.5)Al, 88.7(5.5)Si-2.5(1.4)Nb-2.4(1.2)Ti-3.6(2.3)Cr-2.8(0.8)Al, and 97(0.8)Al-2.4(0.7)Si-0.3Cr-0.2Ti-0.1Nb. The (Si)_ss_ was not confirmed by XRD owing to its low volume fraction in the alloy.

The bulk microstructure was coarser than those in the bottom and top of the button and there was a transition from the bottom to the bulk (TFBTB), see Figure 5. The OHC5-AC could be considered to have a “layered” structure. Elongated facetted dendrites were formed and there was some porosity. The typical microstructure in the bulk of the button is shown in the Figure 5a,b. It consisted of facetted (Nb,Ti)(Si,Al)_2_ dendrites with small inter-dendritic regions that were composed of the (Cr,Ti,Nb)_6_Si_5_, and (Cr,Ti,Nb)(Si,Al)_2_ compounds and a very low volume fraction of (Si)_ss_ that exhibited black contrast. The microstructures in the top and TFBTB were similar. Details of the microstructure in the bottom of the button are shown in the Figure 5c. The (Nb,Ti)(Si,Al)_2_, (Cr,Ti,Nb)_6_Si_5_, (Cr,Ti,Nb)(Si,Al)_2_ compounds were still present but in this part of the button the (Si)_ss_ was not found, instead the (Al)_ss_ was observed. The (Nb,Ti)(Si,Al)_2_ dendrites were thinner and the inter-dendritic regions larger than in the bulk. There was microsegregation in (Cr,Ti,Nb)_6_Si_5_ in which the concentrations of Nb and Cr were the highest respectively in the bulk (about 23 at.%) and edge (about 27 at.%) and the lowest respectively in the edge (about 6 at.% Nb) and centre (about 13 at.% Cr) of grains. There was also microsegregation in (Nb,Ti)(Si,Al)_2_ in the top and bottom of the button but not in the bulk. The DSC trace (not shown) exhibited an endothermic peak starting at 569 °C. This was attributed to the melting of the (Al)_ss_.

### 4.2. Heat Treated Alloys

**OHC1**: The actual composition (at.%) of the heat treated alloy (OHC1-HT, 1200 °C/48 h) was Si-22.1Fe-15.1Cr-15.8Ti-1.1Nb. This was the average value of the large area analyses taken from all parts of the button. The microstructure had coarsened and consisted of the same phases as OHC1-AC (Figure 1a). The volume fraction of (Fe,Cr,Ti)Si had decreased and the volume fractions of the FeSi_2_Ti and (TM)_6_Si_5_ had increased. In OHC1-HT it was more noticeable that the (Fe,Cr,Ti)Si phase surrounded (enveloped) a thin layer of the FeSi_2_Ti. The Si and Ti contents of the FeSi_2_Ti increased by 9% and 49% respectively, bringing its average composition (50.2Si-25Fe-3.3Cr-20.9Ti-0.6Nb) very close to that reported in [39]. The compositions of (Fe,Cr,Ti)Si and (TM)_6_Si_5_ essentially were the same as in OHC1-AC. The (TM)_6_Si_5_ had cracks and pores, and chemical inhomogeneity was still present. 

**OHC5**: After the heat treatment at 1400 °C for 100 h, the actual composition of OHC5-HT was Si-26.2Nb-4.9Cr-5.2Ti-3.8Al. This was the average of the analyses taken from all parts of the button. Chemical inhomogeneity was still present. Liquation in the specimen or staining of the crucible after the heat treatment were not observed. By liquation it is meant that there was no noticeable distortion of the shape of the heat treated cube. The (Al)_ss_ observed in OHC5-AC would be expected to melt at this temperature.

**Figure 6:**
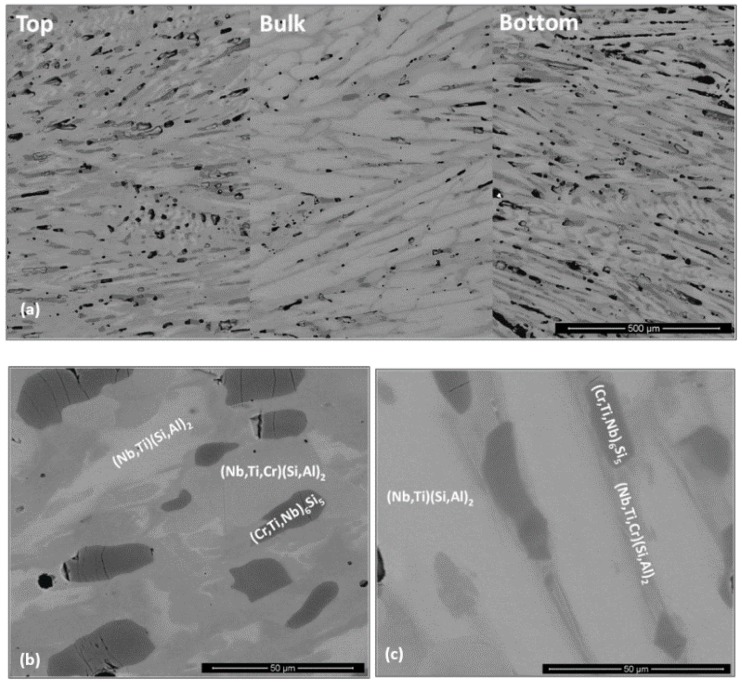
BSE images of the microstructure of the alloy OHC5-HT, (**a**) cross section, ×200 (**b**) top, ×2000 and (**c**) bulk (×2500) of the button.

The microstructure (Figure 6) consisted only of two phases, namely the (Cr,Ti,Nb)_6_Si_5_ embedded in a matrix of (Nb,Ti)(Si,Al)_2_. This was confirmed by the XRD (Figure 1b). Owing to the dissolution of the (Cr,Ti,Nb)(Si,Al)_2_, two compositions were identified for the Nb rich disilicide, namely (Nb,Ti)(Si,Al)_2_ (62.1(0.5)Si-31.1(0.9)Nb-2.1(0.6)Ti-3.9(0.5)Al-0.9Cr) and (Nb,Ti,Cr)(Si,Al)_2_ (62(0.5)Si-26(1.1)Nb-5.6(0.8)Ti-2.3(0.4)Cr-4.1(0.5)Al. In the (Cr,Ti,Nb)_6_Si_5_ (45.3(0.2)Si-10.7(1.3)Nb-15.1(1)Ti-28.3(1.7)Cr-0.7Al) cracks were observed, its Cr concentration had increased to 28 at.% Cr and its Al content was practically negligible.

The chemical inhomogeneity in the (Nb,Ti)(Si,Al)_2_ compound was more evident in the top and bottom than in the bulk, and its darker contrast areas were Cr-rich. There was an increase of the volume fraction of (Nb,Ti,Cr)(Si,Al)_2_ and (Cr,Ti,Nb)_6_Si_5_. The concentration of transition metals in the latter had slightly changed compared with OHC5-AC. In the top of the button its Ti content was essentially fixed at 14 at.%, but in the bottom it was in the range 15.3 at.% to 17 at.%. The typical microstructure in the bulk of the heat treated button (Figure 6c) consisted of (Nb,Ti)(Si,Al)_2_ matrix with coarsened (Cr,Ti,Nb)_6_Si_5_ and a very low volume fraction of (Nb,Ti,Cr)(Si,Al)_2_. 

### 4.3. Oxidation

**Figure 7:**
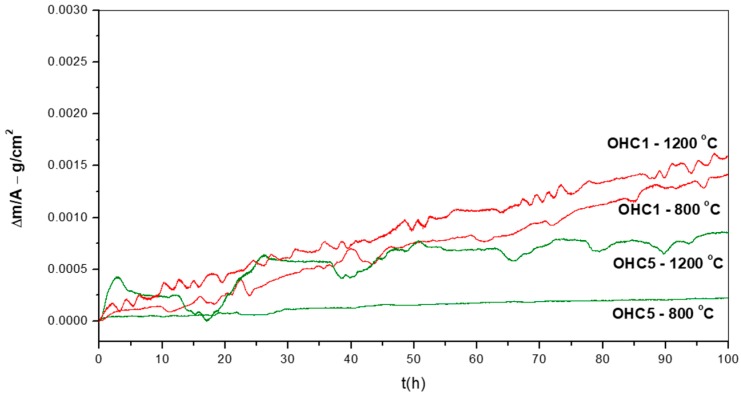
Weight change versus time data for isothermal oxidation at 800 °C and 1200 °C of the alloys OHC1 and OHC5.

The TGA data was analysed using the equation ln(Δw) = lnK + nlnt, where $\Delta w\;=\;\frac{\Delta m}{A}$ and Δw is the weight change per unit area, K is the reaction rate constant that embodies the sum of reaction rates, Δm is the weight change, A is the surface area before exposure and t is the exposure time. This equation was used to determine the mechanism that controlled the oxidation. The oxidation kinetics are regarded as linear (n = 1), parabolic (n = 0.5), sub-parabolic or cubic (n ≤ 0.3). If there was more than one mechanism involved, the corresponding section was evaluated to determine the oxidation kinetics from the equation $\Delta w\;=\;k_{l}\cdot t$ for linear oxidation and ${\left(\Delta w\right)}^{2}\;=\;k_{p}\cdot t$ for parabolic oxidation, where k_l_ is the linear rate constant and k_p_ is the parabolic rate constant [41]. The oxidation data of the two alloys (Figure 7) is summarised in Table 1. The alloy OHC1 gained more weight than OHC5 (Table 1). Both alloys did not pest at 800 °C. 

**OHC1-800 °C**: The cubic specimen had retained its shape and had sharp edges; its surface was slightly lustrous with some greenish and golden tones. In the early stages of the oxidation and before the isothermal temperature was reached red rust like staining was found on the contact surface of the alumina crucible with the specimen and remained until the experiment was finished. This suggested that a chemical reaction of fast growing oxide(s) with alumina occurred at the beginning of the experiment but did not continue during the isothermal oxidation. The oxidation data gave n = 0.89, in the first 10 h the oxidation was parabolic and for the rest of the experiment linear (Table 1). Figure 7 shows repeated periods of weight loss after gain weight. The total time of weight loss was 19 h. The total time that the sample gained weight was 81 h of which 71 h was with linear and 10 h with parabolic oxidation kinetics. In the first 10 h the oxidation was parabolic. This was attributed to the formation of SiO_2_ (see below), which is the most protective oxide that this alloy could form. No oxide spallation was observed but there were some cracks on the surface of the scale. These cracks could have been caused by volume changes resulting from phase transformations due to the selective oxidation of the alloy’s components and/or stresses arising from the growth of oxide(s).

**Figure 8:**
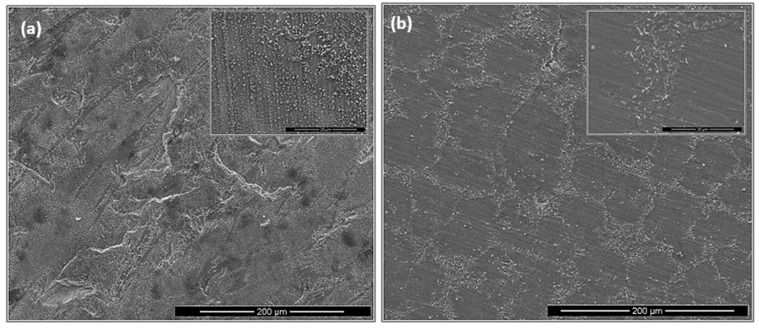
SEM images of alloy OHC1 after isothermal oxidation in air at 800 °C for 100 h, scale (**a**) parallel, ×500 and (**b**) normal (×600) to the dendritic growth of the (TM)_6_Si_5_.

The scale was very thin, brittle and easy to spall off during sample preparation, which made difficult the characterization of cross sections. Figure 8 shows the scale on two sides (facets) of the cubic specimen after oxidation. On both sides an adherent and continuous scale were formed that consisted of a continuous glassy like layer, and regions with a dispersion of fine faceted particles (see inserts). Also, cavities were observed. The scale exhibited different characteristics in the two sides that were attributed to the orientation of the underlying phases in the alloy (substrate). One side of the specimen presented higher volume fraction of fine granular particles in the continuous glassy oxide over the TM_6_Si_5_ phase (Figure 8a) while the (Fe,Cr,Ti)Si phase was covered by the continuous glassy oxide layer (Figure 8a). The other side of the specimen (Figure 8b) shows the oxide that formed perpendicular to the dendrites of the (TM)_6_Si_5_ phase and had a lower volume fraction of the granular particles.

The scale over the (TM)_6_Si_5_ phase depended on the microsegregation of Fe in this phase (Figure 2). Indeed, the granular oxide formed on this phase was coarser in the Fe rich areas (edges). Some porosity also was observed in the scale in the centre of the (TM)_6_Si_5_ phase that could be attributed to oxide evaporation; these areas were richer in Nb, Cr and Ti (Figure 2). Black areas in Figure 8 were due to excess C deposition for sample preparation.

**Figure 9:**
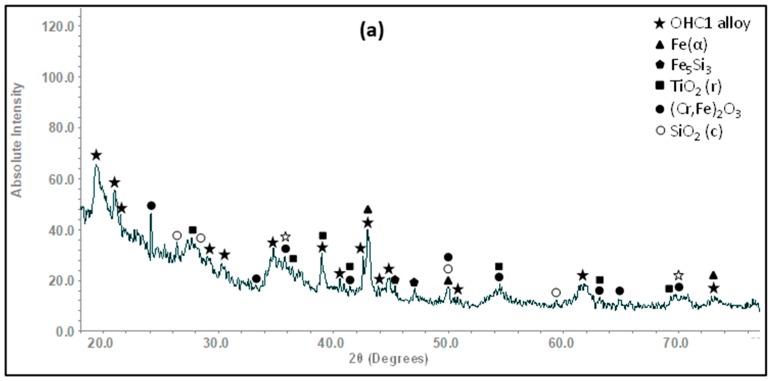

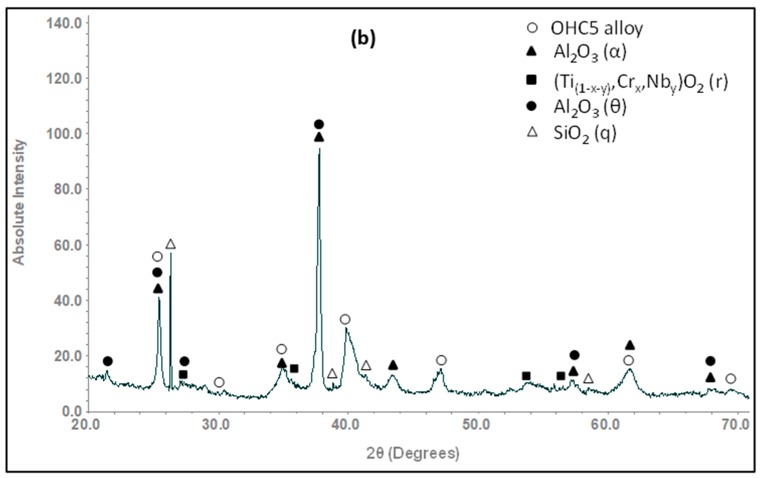
GXRD data of the scale formed on the alloys (**a**) OHC1 (θ = 1°) and (**b**) OHC5 (θ = 5°) at 800 °C.

In the diffusion zone there was Si depletion in all the phases owing to the growth of SiO_2_. Near the substrate/scale interface this Si depletion was more noticeable leading to transformation(s) to phase(s) richer in transition metals. In the GXRD data (Figure 9a), some peaks from the alloy (substrate) were present. These were mainly from the FeSi_2_Ti and (TM)_6_Si_5_ phases. There were also peaks corresponding to the Fe_5_Si_3_ and Fe(α) phases. The latter were the result of phase transformation(s). The oxide peaks corresponded to SiO_2_ in the form of cristobalite (JCPDS 39-14250), (Cr,Fe)_2_O_3_ (JCPDS 02-1357) and TiO_2_ (rutile) (JCPDS 89-4920). According to the EDS data, (Ti,Cr)O_2_ and/or (Ti,Cr,Nb)O_2_ could be present in the scale depending on the composition of the underlying phase in the substrate.

**Figure 10:**
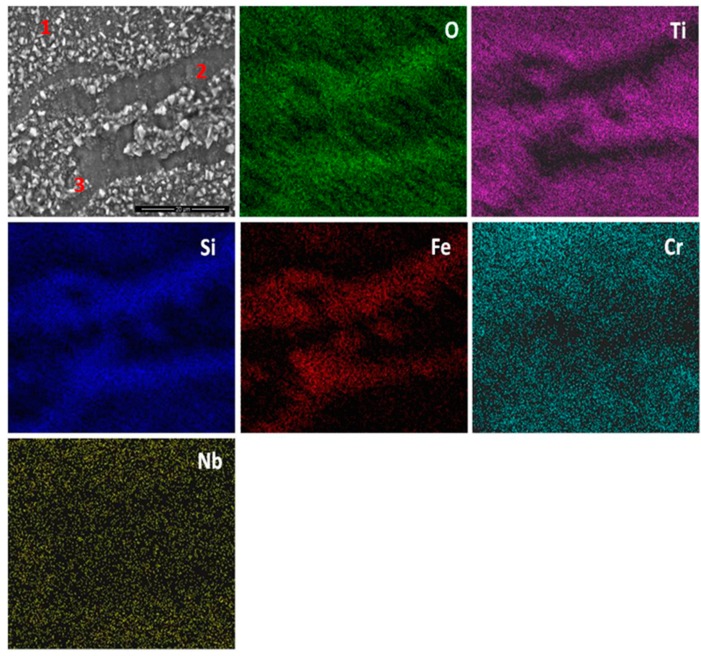
EDS X-ray elemental maps of the scale of the alloy OHC1 after isothermal oxidation at 800 °C for 100 h. BSE image ×4000. The EDS spectra of qualitative point analyses at 1, 2 and 3 were, respectively Si rich with Ti,Cr,Nb and Fe, Si rich with Fe and Cr, and Ti rich with Si.

The elemental X-ray maps of the scale are shown in Figure 10. Considering the GXRD data in Figure 9a and the Figure 10, SiO_2_ and possibly some Fe_2_O_3_ formed over the (Fe,Cr,Ti)Si phase, coarse granular particles over the FeSi_2_Ti phase were composed of a Ti-rich oxide enriched by Cr, perhaps with some Si, and over the (TM)_6_Si_5_ some SiO_2_ formed together with Ti rich-oxide with some Cr and Nb enrichment and some (Cr,Fe)_2_O_3_ oxides.

**Figure 11:**
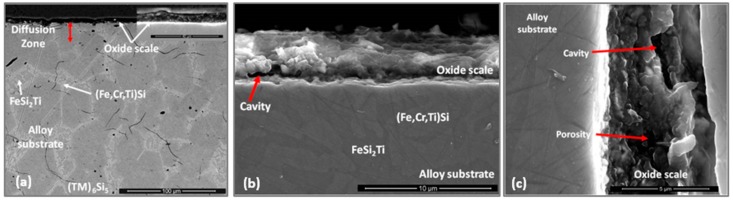
SEM images of the cross section of the alloy OHC1 after isothermal oxidation at 800 °C for 100 h, (**a**) BSE, ×1000 and (**b**) SE (×12,000) images of the scale/metal interface, (**c**) SE image (×20,000) showing cavity and porosity in the scale.

The qualitative chemical analysis (Figure 10) confirmed that continuous SiO_2_ was present all over the alloy but at different volume fractions depending on the oxidised phase and the dominant oxide. This analysis was not conclusive because it also included data from phase(s) that were beneath the scale. The cross section shown in Figure 11a depicts the thickness of the scale and shows that there was a minimum alloy recession. There were some areas in the substrate/scale interface that showed cracks possibly due to embrittlement. The scale was composed of different oxides. The thickness of the scale was in the range 1 to 6 μm because of the different oxidation rates of the phases of this alloy. Figure 11b shows the scale integrity. While some areas were covered by a continuous oxide that enveloped granular particles, some other areas presented cavities in the substrate/scale interface (Figure 11c).

**Figure 12:**
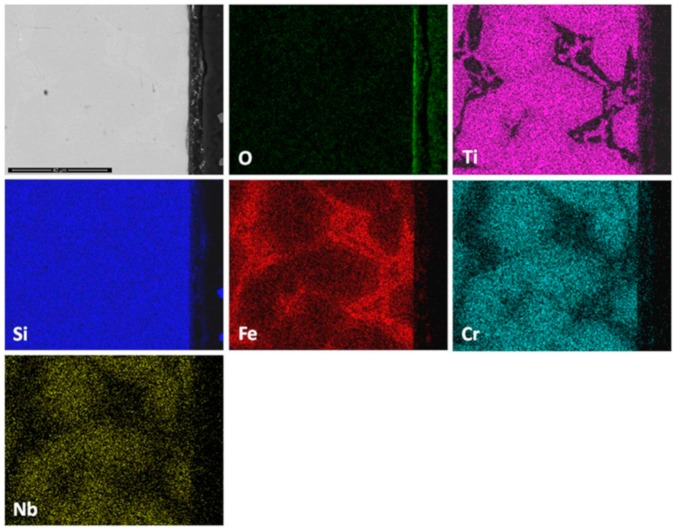
EDS X-ray elemental maps of a cross section of the alloy OHC1 after isothermal oxidation at 800 °C for 100 h, BSE image ×3500.

There was no evidence of internal oxidation in the alloy. In Figure 12 can be seen the facetted hexagonal cross section of (TM)_6_Si_5_ dendrites with edges defined by the (Fe,Cr,Ti)Si phase, and the latter surrounding the FeSi_2_Ti phase (Figure 3). The scale was Si rich and contained Ti, Fe and Cr. It is not easy to reproduce the contrast from the Si pixels in the Si map. The contrast of the phases did not change significantly near the substrate/scale interface, and the elemental X-ray map did not show changes in Si concentration. In the diffusion zone there was some Si depletion in all the phases due to oxidation that resulted to the (Fe,Cr,Ti)Si and FeSi_2_Ti compounds becoming richer in Fe. The Si depletion of the (Fe,Cr,Ti)Si led to the formation of Fe_5_Si_3_ and Fe(α) at the substrate/scale interface, and in the case of FeSi_2_Ti led to the formation of τ_3_ (Fe_52_Si_36_Ti_12_ or Fe_4_Si_3_Ti [42]) and Fe(α). There was also Si depletion from the (TM)_6_Si_5_, but not enough to trigger a phase transformation. The Si depletion at the substrate/scale interface and the mechanical damage to the SiO_2_ layer as a result of the development of strains from volume changes due to phase transformations could have led to further oxidation and the formation of mixed oxides. It is possible that within the scale more strains could arise as oxides with different volumes formed, and that further variations in chemistry affected their volume during growth.

**OHC5-800 °C**: Figure 7 shows the isothermal oxidation data. There were three oxidation stages, the first lasted 1.3 h, the second 22.7 h and the third to the end of the experiment. The oxidation was parabolic (n = 0.54) and was composed of slightly different parabolic oxidation rates (Table 1) that could be attributed to the nature of the oxides and any phase transitions that could have occurred during oxidation. This behaviour could be associated with some type of transient oxidation in the first 24 h giving a slow growth scale that possibly cracked and spalled off before a more protective scale was stablished. Phase transitions and chemical reactions have been linked with changes of oxidation rate [43]. 

**Figure 13:**
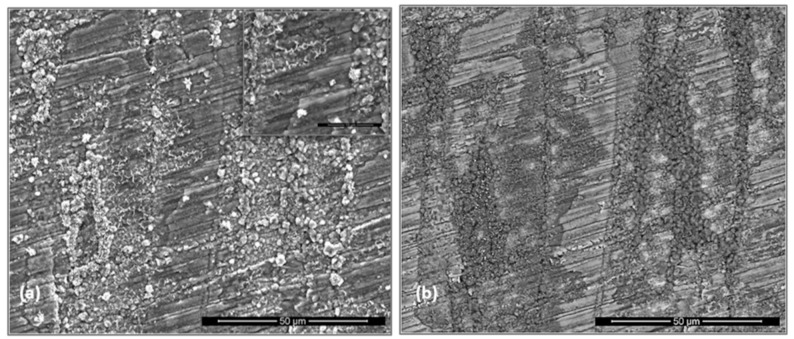
SEM images of the scale formed on the alloy OHC5 after isothermal oxidation at 800 °C for 100 h, (**a**) SE image, ×2000 with insert at ×8000 (**b**) BSE image, ×2000.

**Figure 14:**
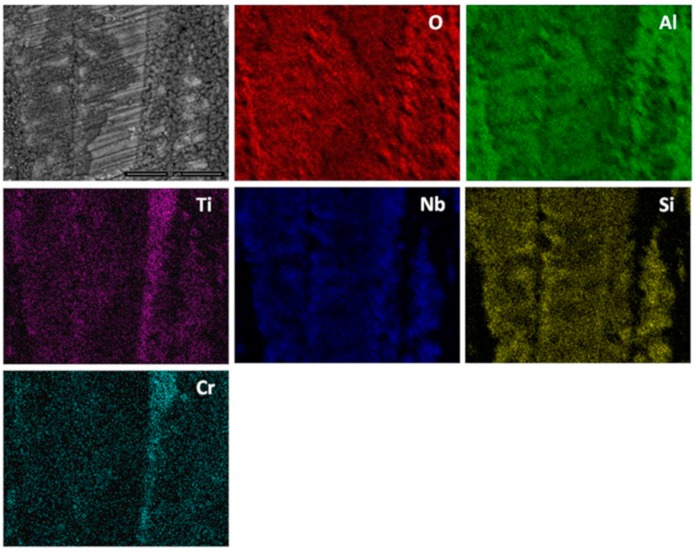
EDS X-ray elemental maps of the scale of the alloy OHC5 after isothermal oxidation at 800 °C for 100 h, BSE image ×4000.

The cubic specimen remained intact with well-defined sharp edges; its surface was covered by a black oxide layer. An adherent scale had formed and different microstructures had resulted from the oxidation of the underlying phases in the substrate (Figure 13). The lumpy areas were composed of clusters of angular oxide particles, ridge network like areas were formed over the Al rich areas in the substrate, and thin, flat and continuous oxide was observed over the rest of the alloy giving bright contrast over the (Nb,Ti)(Si,Al)_2_ and grey contrast over the (Cr,Ti,Nb)_6_Si_5_ phases. The GXRD data in Figure 9b suggested that the scale consisted of α-Al_2_O_3_ (JCPDS No. 10-173), θ-Al_2_O_3_ (JCPDS No. 50-1496), quartz SiO_2_ (JCPDS No. 47-1144), and rutile TiO_2_ (JCPDS No. 21-1276). Rutile type complex oxides like (Ti_(1−x−y)_,Cr_x_,Nb_y_)O_2_ could be present in the scale. Although the scale was mainly composed of Al and O, some strong signals from the transition metals were also visible. Figure 14 shows the Al_2_O_3_ in different regions and the regions where the Cr, Nb and Ti were the main components suggesting the formation of a scale of Al_2_O_3_ and some SiO_2_.

**Figure 15:**
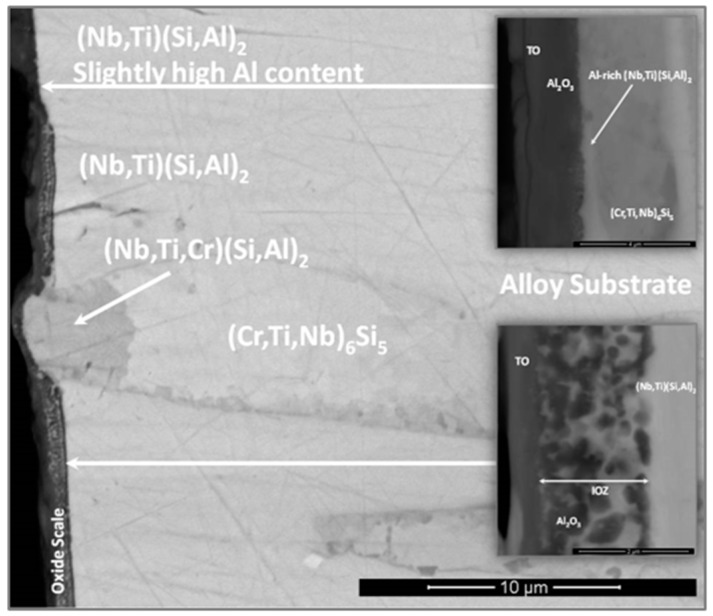
BSE images of a cross section of the alloy OHC5 after isothermal oxidation at 800 °C for 100 h. Main image, ×7000, top insert, ×30,000 and bottom insert, ×60,000.

The thickness of the scale was in the range of 1 μm to 4 μm (Figure 15) and depended on the underlying phase. The scale that formed on top of the (Nb,Ti)(Si,Al)_2_ was characterised by two different microstructures, both presented a transition oxide (TO) at the oxide/gas interface, which, according to chemical analyses, was composed of all the components, one presented an internal oxidation zone (IOZ) composed of Al_2_O_3_ particles dispersed in the (Nb,Ti)(Si,Al)_2_ followed by a thin and continuous layer of Al_2_O_3_ at the scale/substrate interface, and another that did not present an IOZ but a continuous Al_2_O_3_ layer up to the substrate/scale interface. The latter was also slightly enriched in Al at grain boundaries, which is consistent with the microsegregation in the (Nb,Ti)(Si,Al)_2_ phase, that was richer and leaner in Ti, Al and Cr respectively at the grain boundaries and in the middle of the grains. The IOZ in the (Nb,Ti)(Si,Al)_2_ phase was formed in the areas where the Al content at the substrate/scale interface was below 3 at.% Al. The (Cr,Ti,Nb)(Si,Al)_2_ was detected in the ridges at the substrate/scale interface. The scale formed on top of these areas was thinner with high Al and Si content. The (Nb,Cr,Ti)_6_Si_5_ phase formed a very thin oxide (Figure 15). 

**Figure 16:**
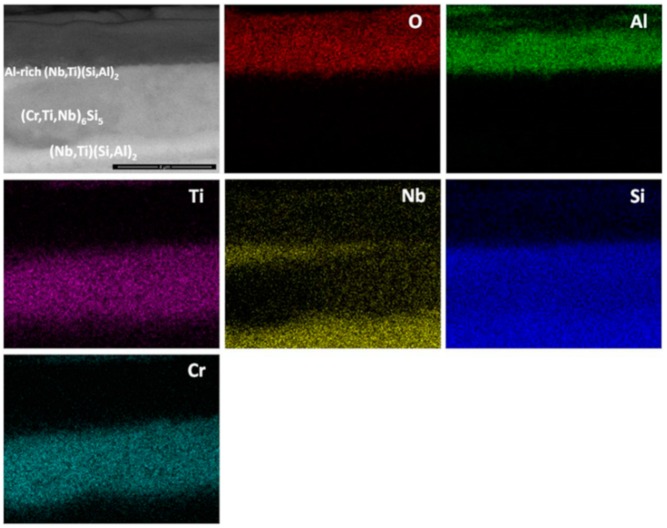
EDS X-ray elemental maps of a cross section of the alloy OHC5 after isothermal oxidation at 800 °C for 100 h, BSE image ×30,000.

Al and O were the main components of the scale (Figure 16). Some Nb, Ti, Cr and Si were also found at the gas/oxide interface. Considering Figure 14 and Figure 16, it is possible to locate the rutile type oxides which were mostly found at the scale/gas interface with a significant Al_2_O_3_ content. In particular, Figure 16 shows that Al_2_O_3_ was the oxide that formed on top of the (Nb,Ti)(Si,Al)_2_ phase.

**OHC1-1200 °C**: Overall the oxidation was para-linear, as both parabolic and linear mechanisms had occurred [41]; in the first 40 h the oxidation was parabolic and after this time it changed to linear. It was not possible to get the n value. However, it was possible to evaluate the rate constants for different periods during the isothermal oxidation (Table 1). The oxidised specimen had retained its shape and had sharp edges. The scale had good adherence. Layering, voids and discontinuities were visible in the scale. The latter was mainly composed of agglomerated particles of Cr_2_O_3_ of different sizes that did not form a continuous scale, showing discontinuities, possibly as a result of oxide evaporation, porosity and cracks resulting from the formation of different oxides. The areas where SiO_2_ was formed with a glassy like appearance were mostly found at the grain boundaries between the (Fe,Cr,Ti)Si and FeSi_2_Ti phases.

**Figure 17:**
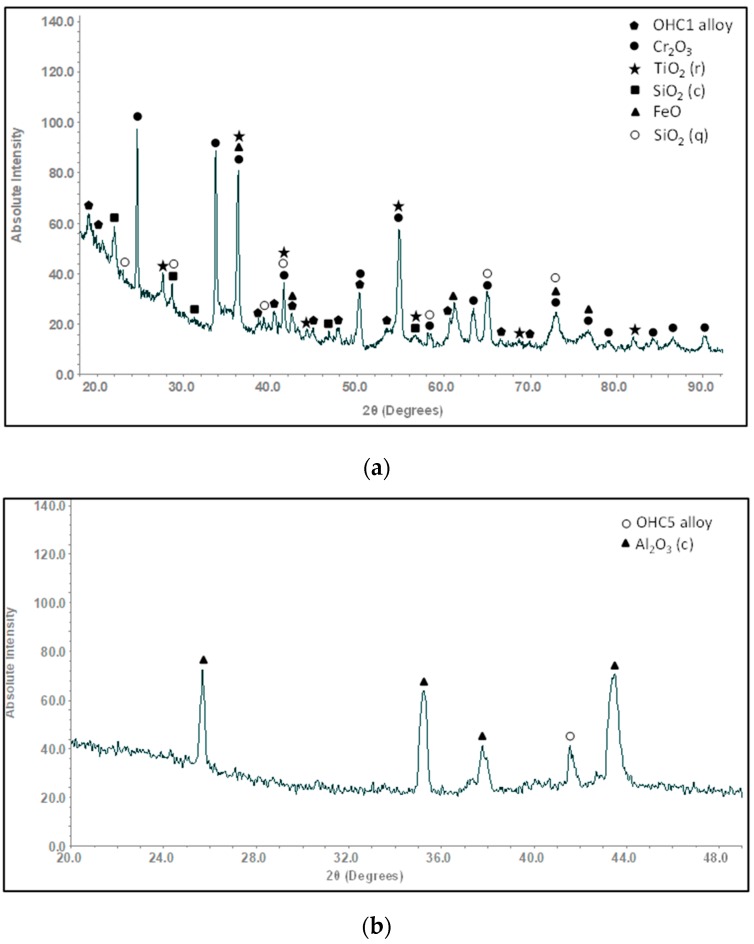
GXRD data of the scale formed on the alloys (**a**) OHC1 (θ = 10°) and (**b**) OHC5 (θ = 5°) at 1200 °C.

**Figure 18:**
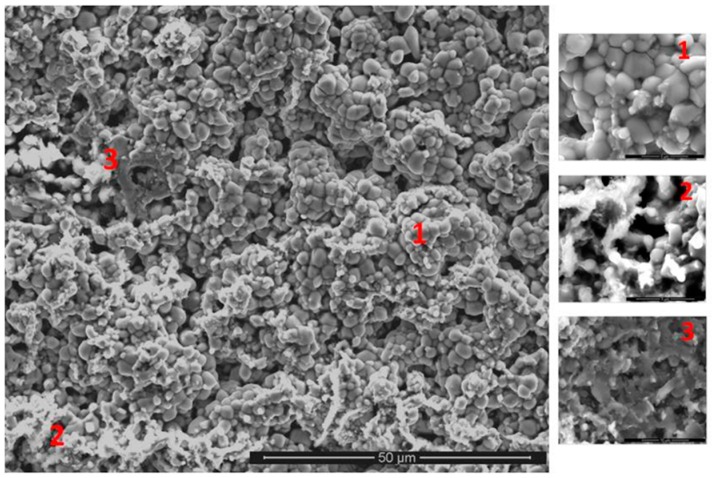
SE images of the scale of the alloy OHC1 after isothermal oxidation at 1200 °C for 100 h, ×1500. In the right-hand side are shown the microstructures of different areas of the scale (1) ×8000, (2) ×16,000 and (3) ×20,000.

The GXRD data (Figure 17a) confirmed the presence of Cr_2_O_3_ (JCPDS 38-1479), at least two crystalline forms of SiO_2_ (cristobalite JCPDS 39-1425, and quartz JCPDS 70-2537), TiO_2_ (JCPDS 84-1284) and FeO (JCPDS 06-0615). Figure 18 shows the three typical morphologies of the oxides that composed the scale. The insert image 1 shows the regions composed of granular oxide particles with different sizes that formed on top of the (TM)_6_Si_5_ phase. These were present with a higher volume fraction. The regions that corresponded to the image 2 were found in contact with areas covered by finer particles of an oxide with a glassy appearance on the top. The insert image 3 is typical of some areas in which the microstructure looked more like a network formed by the melting of some oxide, this was mainly observed on top of the (Fe,Cr,Ti)Si phase.

**Figure 19:**
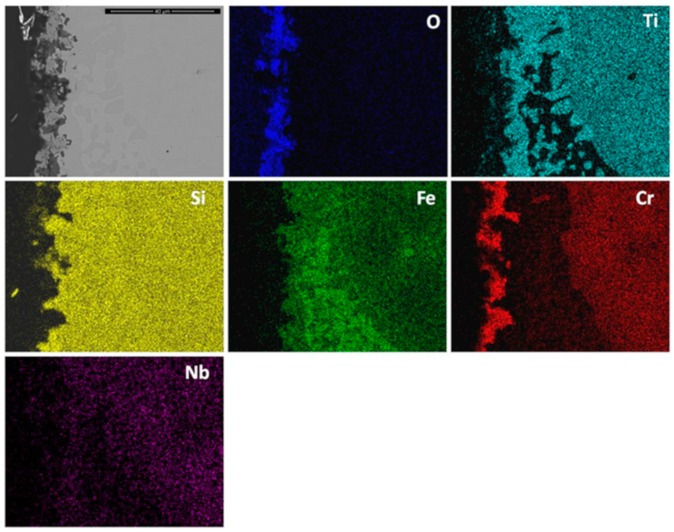
EDS X-ray elemental maps of a cross section of the alloy OHC1 after isothermal oxidation at 1200 °C for 100 h, BSE image ×3500.

Cross sections of the scale showed that it was mainly composed of two layers, namely the Cr_2_O_3_ that was mostly found in the outer part of the scale and the SiO_2_ in the inner part (Figure 19). (Cr,Ti)_2_O_3_ was also found at the substrate/scale interface. In these areas there were some voids possibly due to metal transport through the scale. The substrate below the scale was composed of the FeSi_2_Ti and (Fe,Cr,Ti)Si phases, which would suggest that the (TM)_6_Si_5_ phase had transformed to FeSi_2_Ti when the Cr was preferentially oxidized and the (Fe,Cr,Ti)Si phase was oxidized and formed Si and Fe oxides. There was also evidence of internal oxidation with Si rich oxide particles distributed not randomly. The presence of Ti, Fe and Nb (if any) in the scale was weak (Figure 19), suggesting that Ti and Fe could be in solution in Cr_2_O_3_ (Fe_2_O_3_ and Cr_2_O_3_ have the same crystal structure). The Nb could be in solution in the TiO_2_ phase [5].

**Figure 20:**
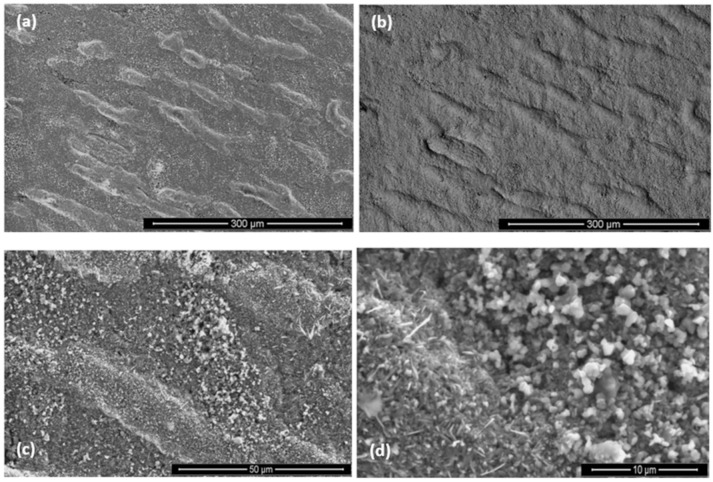
SEM images of the surface of the scale of the alloy OHC5 after isothermal oxidation at 1200 °C for 100 h, (**a**) SE image, ×200, (**b**) BSE image, ×200, (**c**) SE image, ×1000 and (**d**) SE image, ×3500.

**OHC5-1200 °C**: The cubic specimen had remained intact with well-defined and sharp edges; its surface was covered by a light green colour scale. In the scale there was porosity (Figure 20a) and partial spallation that did not expose the substrate. There were also ridges on the scale surface. The scale spallation was mostly found next to oxide lumps. Figure 20b would suggest that the scale consisted of only one oxide, since an even contrast was observed under BSE imaging conditions. The secondary electron (SE) images in Figure 20c and d illustrate an adherent scale that was composed of a mixture of needle-like and facetted granular particles. Under BSE imaging these oxide particles did not show different contrasts. The GXRD data in Figure 17b suggested that the scale consisted of Al_2_O_3_ corundum (JCPDS 10-173). The different morphologies of the alumina particles in the scale could be the result of a sluggish transformation from some of its metastable forms. The corundum type of Al_2_O_3_ is the stable form above 1100 °C. However, it is possible that some trace amounts of transition aluminas could have been retained. The peaks from the substrate corresponded to the (Cr,Nb,Ti)_6_Si_5_ phase (JCPDS 54-0381).

As was the case for the oxidized alloy at 800 °C, the oxide lumps were located where the transformation from (Cr,Ti,Nb)(Si,Al)_2_ to (Nb,Ti,Cr)(Si,Al)_2_ had occurred. It is possible that this transformation influenced the size of the oxide particles, and resulted to a finer grain size in these areas compared with the rest of the alloy. Some scale spallation occurred mainly over the inter-dendritic areas. The excess of Al and Cr in such areas could promote a faster scale growth increasing the strain in the scale.

**Figure 21:**
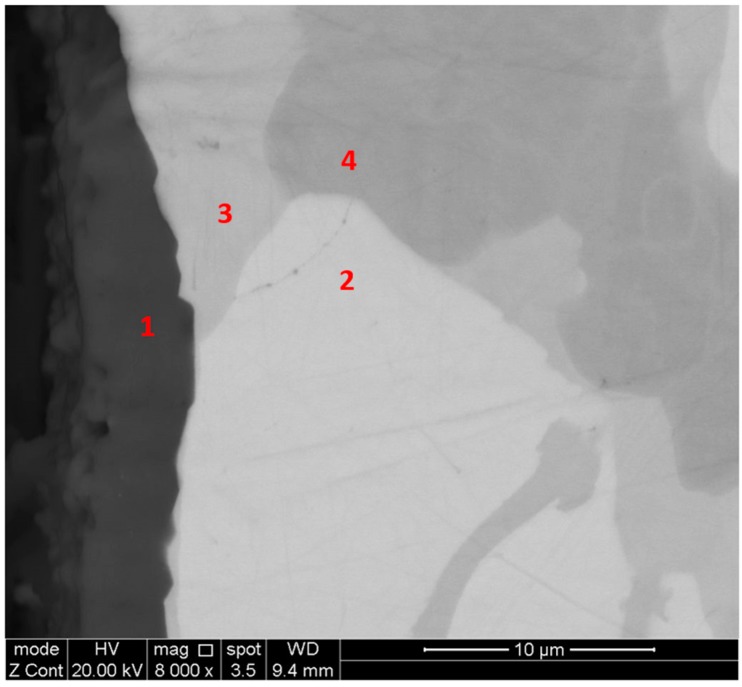
BSE image (×8000) of a cross section of the alloy OHC5 after isothermal oxidation at 1200 °C for 100h. The EDS qualitative analyses spectra for 1 to 4 indicated (1) Al_2_O_3_, (2) (Nb,Ti)(Si,Al)_2_, (3) (Nb,Ti,Cr)(Si,Al)_2_ and (4) (Nb,Cr,Ti)_6_Si_5_.

**Figure 22:**
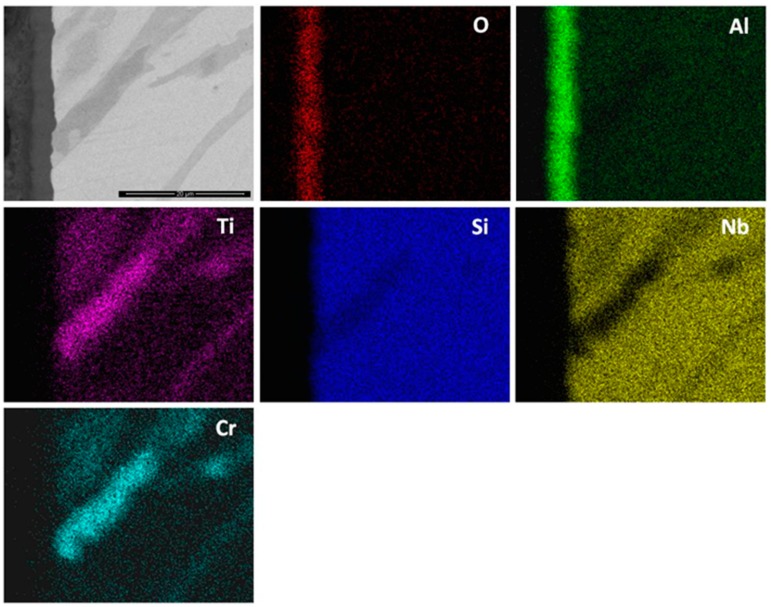
BSE image (×6000) and EDS X-ray elemental maps of a cross section of the alloy OHC5 after isothermal oxidation at 1200 °C for 100 h.

The scale was continuous and compact and its thickness was in the range 5 to 10 μm depending on the underlying phase (Figure 21). At 1200 °C only the (Nb,Ti)(Si,Al)_2_, (Nb,Ti,Cr)(Si,Al)_2_ and (Cr,Nb,Ti)_6_Si_5_ phases were stable. The scale over all the phases of the substrate was composed of Al and O (Figure 22). Even the (Cr,Nb)_6_Si_5_ phase with its low Al content was able to form some Al_2_O_3_. There was no significant Al depletion in the (Nb,Ti)(Si,Al)_2_ silicide, instead this phase was found to be richer in Al and Cr at the substrate/scale interface, possibly due to the dissolution of the (Cr,Ti,Nb)(Si,Al)_2_ phase. The grain boundary areas were richer in Al and Cr in the places where the (Cr,Ti,Nb)(Si,Al)_2_ phase could have dissolved and in these areas the (Cr,Nb,Ti)_6_Si_5_ phase was found to be richer in Cr by 63%. A thicker Al_2_O_3_ scale was also observed in these areas. This would suggest that the grain boundaries played an important role in the oxidation of the alloy OHC5 at this temperature.

## 5. Discussion

### 5.1. Microstructures

**OHC1**: In the top and bulk of OHC1-AC the T ((TM)_6_Si_5_), τ_1_ (FeSi_2_Ti) and (Fe,Cr,Ti)Si compounds were observed. The latter two were formed in-between the T phase dendrites, but it was not clear whether the τ_1_ (FeSi_2_Ti) was surrounded by the (Fe,Cr,Ti)Si, which would be consistent with a peritectic reaction, or formed lamellae next to the (Fe,Cr,Ti)Si, which would be consistent with the eutectic L → τ_1_ (FeSi_2_Ti) + (Fe,Cr,Ti)Si that was observed in the bottom and chill zone of the button (Figure 3b,c). 

A peritectic reaction would explain some of the microstructures shown in the Figure 3a but not the peritectic reaction L + τ_1_ (FeSi_2_Ti) → FeSi + τ_4_ (Fe_28.1_Ti_26.3_Si_45.6_) suggested by Weitzer et al. [39] because the presence of the τ_4_ (Fe_28.1_Ti_26.3_Si_45.6_) was not confirmed by XRD. Some of the analyses that were designated to the T phase corresponded to the composition of the τ_4_ phase. It could be argued that, owing to the partitioning of solutes, some τ_4_ was actually present near the (Fe,Cr,Ti)Si. However, if the aforementioned peritectic reaction had occurred one would expect it to move towards completion upon heat treatment, which means that the size and volume fraction of the τ_1_ (FeSi_2_Ti) would decrease and the size and volume fraction of (Fe,Cr,Ti)Si would increase after the heat treatment. Exactly the opposite was observed. 

In the Cr-Ti-Si system [40] the T phase is stable below 1565 °C and in the Fe-Ti-Si system [39] the τ_1_ (FeSi_2_Ti) is stable below 1532 °C and the FeSi below 1328 °C. Alloying the latter with Cr would be expected to increase only slightly the above temperature. The alloying with Ti would not raise the melting temperature of (Fe,Cr,Ti)Si above 1532 °C (in the Si rich region of the Fe-Ti-Si system the TiSi is stable below 1450 °C). 

The formation of the T phase was accompanied by the partitioning of Fe and Cr, Nb and Ti. Iron was rejected into the melt, while the other elements partitioned in the solid, see Figure 2. Thus, as the T phase was formed the surrounding melt became richer in Fe and leaner in Cr, Nb and Ti. The τ_1_ (FeSi_2_Ti) + (Fe,Cr,Ti)Si eutectic that was formed in-between T phase dendrites was richer in Fe and lean in Nb, Ti and Cr compared with the alloy composition. 

The formation sequence, in terms of decreasing temperature, of the intermetallic phases in OHC1-AC should be T, then τ_1_ (FeSi_2_Ti) and finally (Fe,Cr,Ti)Si. It is suggested that the solidification path was L → L + T → T + {τ_1_ + (Fe,Cr,Ti)Si}_eutectic_ with a very small volume fraction of τ_1_ in the top and bulk of the button owing to the composition of the inter-dendritic melt relative to the eutectic composition. According to the data in [39], the solubility range of τ_1_ (FeSi_2_Ti) is very narrow, which could be another reason for its difficulty to form in the top and bulk of OHC1-AC. Indeed, the composition of this phase moved closer to the one reported by Weitzer et al. [39] after the heat treatment, owing to the partitioning of solutes in the microstructure.

In the areas near to the bottom and the chill zone of the button, the solidification path was essentially the same as described above but because the melt was richer in Fe and leaner in Cr, Nb and Ti (owing to the macrosegregation in OHC1-AC) the inter-dendritic melt was closer to the eutectic composition and thus the volume fraction of the eutectic was higher in these areas of the button.

In the T phase the partitioning of Ti and Cr was opposite to that of Fe (Figure 2), for the former two elements the partitioning coefficient k_o_^TM^ (TM = Cr, Ti) was greater than one and for Fe it was less than one. Use of the Scheil equation and the concentration profiles of Fe, Cr and Ti in Figure 2 gave k_o_^Fe^ = 0.522, k_o_^Cr^ = 1.482 and k_o_^Ti^ = 1.267. The Ti concentration in (Fe,Cr,Ti)Si was in agreement with Weitzer et al. [39] who reported that the solubility of Ti in FeSi is about 1 at%. 

**OHC5**: In this alloy there was macrosegregation of Al, Cr, Nb, Si and Ti with different profiles of Nb and Si compared with Al, Cr and Ti, and also there was microsegregation, particularly in the (Cr,Ti,Nb)(Si,Al)_2_ and (Cr,Ti,Nb)_6_Si_5_ compounds. The (Nb,Ti)(Si,Al)_2_ was formed at the highest volume fraction and the other intermetallics and solid solutions formed from the liquid between the (Nb,Ti)(Si,Al)_2_ grains. Considering the crystal structures of the binary disilicides NbSi_2_, CrSi_2_ and TiSi_2_ (see Section 2), the former two could form a continuous solution phase. Nakano et al. [44] suggested that very small substitutions of Nb and Si by Ti and Al in NbSi_2_ (with up to 1.7 at.% Ti substituting Nb and up to 2 at.% Al substituting Si) would stabilize the C54 crystal structure. This was not confirmed by our results. Indeed, the TiSi_2_ was not detected in OHC5-AC and OHC5-HT by EDS and XRD. However, the CrSi_2_ was confirmed by XRD (Figure 1) and its Nb, Ti and Al contents were up to 6 at.%, 12.3 at.% and 10 at.%, respectively. The solubility of these elements in the CrSi_2_ based (Cr,Ti,Nb)(Si,Al)_2_ was in agreement with the solubilities reported in the Ti-Cr-Si and Cr-Si-Al ternary systems [40,45].

The ranking of the unalloyed disilicides according to their melting temperatures is T_m_^NbSi2^ = 1935 °C, T_m_^TiSi2^ = 1480 °C and T_m_^CrSi2^ = 1450 °C [19]. The melting temperature of (TM)_6_Si_5_ is higher than 1500 °C (see above). Thus, it would be expected that the primary phase to form from the melt was the intermetallic based on NbSi_2_, namely the (Nb,Ti)(Si,Al)_2_ followed by the (TM)_6_Si_5_ and then the (Cr,Ti,Nb)(Si,Al)_2_ and finally the solid solutions of Si and Al. The primary (Nb,Ti)(Si,Al)_2_ phase formation is supported by the Nb-Cr-Si liquidus projection [46] when the alloy is considered as Cr-(Nb,Ti)-(Si,Al). The formation of the (TM)_6_Si_5_ after the aforementioned primary phase is also in agreement with the liquidus projection. Thus, the solidification path of the alloy OHC5-AC was L →L + (Nb,Ti)(Si,Al)_2_ →L + (Nb,Ti)(Si,Al)_2_ + (TM)_6_Si_5_ → L + (Nb,Ti)(Si,Al)_2_ + (TM)_6_Si_5_ + (Cr,Ti,Nb)(Si,Al)_2_ → (Nb,Ti)(Si,Al)_2_ + (TM)_6_Si_5_ + (Cr,Ti,Nb)(Si,Al)_2_ + (Si)_ss_ or (Al)_ss_ (depending on the solidification conditions and the composition of the last to solidify melt). 

After the heat treatment the (Cr,Ti,Nb)(Si,Al)_2_ and the (Al)_ss_ and (Si)_ss_ were not stable and the Cr concentration in the TM_6_Si_5_ silicide had increased significantly. The former is in agreement with the 1500 °C isothermal section of Cr-Nb-Si [46] when the heat treated alloy is considered as Cr-(Nb,Ti)-(Si,Al) and the latter is attributed to the dissolution of the (Cr,Ti,Nb)(Si,Al)_2_. 

### 5.2. Oxidation

**OHC1-800 °C**: The alloy did not pest. The scale was composed of SiO_2_, TiO_2_ and (Cr,Fe)_2_O_3_. In the substrate below the scale α-Fe, τ_3_ (Fe_40_Si_31_Ti_13_) and Fe_5_Si_3_ were formed owing to the depletion of the elements that formed the oxides in the scale. The location of the oxides in the scale was linked with the underlying phases in the substrate. The microsegregation in the (TM)_6_Si_5_ (Figure 2) affected its oxidation. On top of the (TM)_6_Si_5_ grains the scale was composed of fine granular particles of TiO_2_ engulfed by SiO_2_. The TiO_2_ contained other elements that were in solution in the (TM)_6_Si_5_ (Figure 10); the Cr and Nb concentrations were higher in the centre of the grains and gave the rutile type structure (Ti,Cr,Nb)O_2_ oxide while over the Fe-rich edges of the (TM)_6_Si_5_ no Fe was observed in the oxide owing to the low solubility of Fe in the TiO_2_. The low solubilities of Fe and Cr in TiO_2_, respectively about 1 at.% and 4 at.% [47,48], and the fact that Fe can be transported through SiO_2_ towards the surface of the scale [49] suggested that some (Cr,Fe)_2_O_3_ + TiO_2_ could have formed on top of the Fe-rich areas of the (TM)_6_Si_5_ compound. 

The same oxidation behaviour was observed along (TM)_6_Si_5_ dendrites but in this case the TiO_2_ particles were coarser. The EDS analysis of cross sections of the interface of (TM)_6_Si_5_ with the scale showed a depletion of about 3.5 at.% Si and 2.5 at.% Ti at the substrate/scale interface. Considering the above, and the fact that the volume fraction of (TM)_6_Si_5_ was the highest in the alloy, GXRD was performed at different angles to search for other phases. None was found. 

The X-ray maps (Figure 10) showed Si, Fe and O over the (Fe,Cr,Ti)Si compound. Fe-Si alloys form a sequence of oxide layers depending on their Si content. At low Si concentrations FeO forms next to the bare metal, and engulfs a dispersion of Fe_2_SiO_4_ particles, then follows a layer of Fe_3_O_4_, and finally a layer of Fe_2_O_3_ is formed as the outermost layer. Some internal oxidation of Si has been observed in these alloys [50]. A reduction in the volume fraction of Fe oxides was found in the scale formed on Fe-Si alloys with high Si content in which an inner layer of SiO_2_ and an outer layer of Fe_2_O_3_ were observed [49]. Considering the high Si content of the (Fe,Cr,Ti)Si phase, the latter would be expected to form an inner SiO_2_ layer and Fe_2_O_3_ as the top oxide at 800 °C. It is suggested that these two oxides were formed over the (Fe,Cr,Ti)Si phase since they were confirmed by GXRD (Figure 9a) and Fe, Si and O were present over the (Fe,Cr,Ti)Si phase in the X-ray maps (Figure 10). The EDS analyses revealed that there was mainly Si depletion from the (Fe,Cr,Ti)Si phase that caused the formation of consecutive layers of Fe_5_Si_3_ and α-Fe underneath the scale. The Si depletion was the result of the formation of the SiO_2_ layer. The α-Fe was found at the substrate/scale interface with Si and Cr contents, respectively 17.3 at.% and 3.7 at.%. According to Adachi and Meier [49], this Si concentration is enough to form a continuous SiO_2_ film over this phase. However, they also found some Fe_2_O_3_ at the scale/gas interface that was attributed to Fe transport through the SiO_2_ layer to form Fe_2_O_3_. It is likely that a thin film of (Fe,Cr)_2_O_3_ formed on top of the SiO_2_ that formed on the (Fe,Cr,Ti)Si phase. 

The X-ray maps (Figure 10) showed that on the FeSi_2_Ti phase mainly formed coarse grains of TiO_2_. This is in agreement with the depletion of Ti and Si near the substrate/scale interface and the formation of the τ_3_ (Fe_40_Si_31_Ti_13_) and α-Fe below the scale. The τ_3_ phase was not detected by the GXRD for all the studied glancing angles because the volume fraction of the FeSi_2_Ti phase in the alloy was the lowest.

A comparison of our results with the oxidation of Si-rich Ti containing silicides is reasonable since on the FeSi_2_Ti phase only TiO_2_ and SiO_2_ formed. According to Kofstad [41], the Ti oxidizes more rapidly than Si, thus it is possible that at 800 °C the mobility of metal ions to the surface of this phase was higher that the mobility of Si and this caused the formation of coarse granular TiO_2_ engulfed by a glassy-like SiO_2_ network, see Figure 10. The EDS spectrum (not shown) for point 3 in Figure 10 showed the analysis to be rich in Ti, Si, O and N. In the GXRD diffractograms no nitride peaks were found. However, it is possible that in the earliest stage of oxidation both Ti nitride and TiO_2_ formed, and the N was then released and reacted again with the silicide or trapped under the scale [51,52]. This could explain the formation of pores and cavities beneath and across the scale (Figure 11). The high Fe content of the complex silicides could have increased the mobility of Ti to the surface because coarse grains of TiO_2_ were observed on top of the FeSi_2_Ti phase and the same was observed near the grain boundaries of the (TM)_6_Si_5_ phase, where the Fe content was the highest (Figure 10).

There was some oxidation of the sample before the isothermal oxidation temperature was reached. Some uneven reddish mark was observed on the crucible. It is likely that Fe oxides had reacted with the alumina crucible. The Fe_2_O_3_ has a red colour and the XRD data confirmed the presence of this oxide in the scale. It is unlikely that the reaction with the alumina crucible contributed to the isothermal oxidation because the initial staining on the crucible did not change with time. 

The oxidation data (Figure 7) showed a parabolic weight gain in the first ten hours (Table 1). This may be attributed to the formation of SiO_2_ for which oxidation rates about 10^−13^ g^−2^cm^−4^s^−1^ at 800 °C and about 10^−12^ g^−2^cm^−4^s^−1^ at 950 °C have been reported. The predominant oxidation behaviour was linear after the first 10 h. It is not clear why this was the case, as no oxide spallation was observed, and evaporation of CrO_3_ (in dry conditions) is not expected at this temperature. No significant Cr depletion at the substrate/scale interface was found and there was no extensive Cr_2_O_3_ formation in the scale. It is possible, however, that time-dependent structural changes occurred in the scale that resulted in a linear rate even though diffusion controlled the oxidation. The oxidation of Ti above 700 °C first follows parabolic kinetics (due to oxygen dissolution in the base metal) then changes to linear (after TiO_0.35_ forms as an outer layer where O diffusion is faster) due to a change in the diffusion controlling oxidation mechanism [53]. Moreover, in the temperature range 800–1000 °C, the growth of TiO_2_ scales is characterised by the diffusion of Ti in the inner layer and by the diffusion of oxygen in the outer layer, which creates stress and cracks. TiO_2_ formed at a high volume fraction in the scale, thus it is suggested those changes in the oxidation behaviour of Ti could have had strong influence in the overall oxidation of the alloy.

The coarsening of oxides in the scale could also have been a factor that had an effect on the oxidation behaviour of this alloy. The growth of different oxides of different volumes would give rise to internal stresses and strains in the scale. The strain was released by cracking the scale, thus exposing the substrate to further oxidation. Besides, the depletion of some elements in the alloy near the scale/substrate interface due to oxidation could lead to a phase transformation in these regions, increasing the mismatch at the interphase interfaces, and resulting in strains that changed the adhesion of the scale at the scale/substrate interface. 

**OHC5-800 °C**: The alloy did not pest, instead it followed parabolic oxidation kinetics with n = 0.54 (Table 1) and formed a very thin adherent scale that mainly consisted of Al_2_O_3_. The EDS and GXRD suggested that SiO_2_ and rutile type oxides (Ti_(1−x−y)_,Cr_x_,Nb_y_)O_2_ were also present in the scale. These did not appear to be detrimental to the oxidation resistance of the alloy. Oxides with different morphology and composition (Figure 13 and Figure 14) formed in the scale, which would suggest that it is likely that the oxidation of phase(s) was influenced by their chemical inhomogeneity. 

The phases that were present in the alloy oxidised differently forming a scale of uneven thickness (1–4 μm), Figure 15. The thinner scale was formed on the (Cr,Nb,Ti)_6_Si_5_. The thickness of the scale formed on the (Nb,Ti)(Si,Al)_2_ depended on its Al content, was thinner in those areas where the Al content was above 3 at. % while in the areas with a low Al content (less than 3 at.%) an IOZ formed. In this context not only the scale thickness was affected by the Al content in the (Nb,Ti)(Si,Al)_2_ but also the oxidation mechanism since the development of a different microstructure in the scale was seen (Figure 15). It is highly likely that the local composition of this phase dramatically affected its oxidation. Chemical analyses showed that in areas of Al lean (Nb,Ti)(Si,Al)_2_, the scale consisted of an external layer of transient complex oxides which might not be protective. Thus, Al was possibly internally oxidised until a continuous Al_2_O_3_ layer formed below the IOZ hindering further oxidation. According to Meier and Petit [54], alloys with low solute contents oxidise by inner diffusion of oxygen. On the other hand, near the (Nb,Ti)(Si,Al)_2_ grain boundaries (Al-rich areas) the activity of Al and Si was higher and a scale formed that consisted of an outer layer (transient oxides) mainly composed of Al and Si and an inner layer very rich in Al. The ridges (lumps) at the substrate/scale interface were related to the (Cr,Ti,Nb)(Si,Al)_2_ phase but it is not clear if they had formed as a result of the substrate recession presented by the (Nb,Ti)(Si,Al)_2_ phase (internally oxidised), or by coarsening caused by the loss of Al and Cr from the (Nb,Ti)(Si,Al)_2_ phase or by both phenomena. 

Thus, considering the microstructure of the scale, it is suggested that the oxidation of (Nb,Ti)(Si,Al)_2_ depended on the availability of Al with about 3 at.% Al possibly being the critical content (in the presence of Ti and Cr). Aluminium contents below the critical one would promote a faster inward diffusion of oxygen oxidising Al preferentially inside the phase. This mechanism is consistent with a parabolic behaviour. Above 3 at.% Al, the (Nb,Ti)(Si,Al)_2_ would form an external oxide. The X-ray elemental maps showed Al, O, Nb and Si as the main components of the scale. 

It was expected to find Nb and Si oxides from the oxidation of the (Nb,Ti)(Si,Al)_2_ phase, as it was observed by Zhang et al. [55] and Murakami et al. [56]. The data from our work suggests that the scale formed on this phase was mainly composed of Al_2_O_3_ and that Si and Nb presented a minor contribution but with the same 1:2 ratio as in the NbSi_2_ phase, which suggests that their oxidation in those areas is unlikely to have occurred. Thus, it is proposed that the internal oxidation started from an initial oxidation of all the components where complex rutile oxides with different compositions formed along with SiO_2_ and Al_2_O_3_, the rutile type oxides could have served as a pathway allowing the inward diffusion of oxygen that reacted with Al (not being sufficient to establish a continuous Al_2_O_3_ layer). The scale/substrate interface receded up to an Al_2_O_3_ compact layer that was established below the IOZ. The increase of the oxidation rate could be related to a rapid Al transport through the scale. According to Prescott and Graham [57] θ-Al_2_O_3_ presents a faster Al transport. However, preferential orientation could also influence the Al diffusion towards the substrate/scale interface. 

The thickness of the scale formed on top of the (Nb,Ti)(Si,Al)_2_ compound was dependent on the Cr concentration, as the latter affects the Al activity. It is known that the addition of Cr reduces the concentration of Al required to grow and sustain an alumina scale in Ni-Cr-Al and Fe-Cr-Al alloys during oxidation. Previous studies have shown that a mixed oxide composed by SiO_2_ and Nb_2_O_5_ formed at 750 °C when 8 at.% Cr was added to NbSi_2_, while the addition of 20 at.% Cr improved the oxidation behaviour via the formation of a scale composed by an inner layer of SiO_2_ and an outer layer of Cr_2_O_3_. The underlying substrate alloy was depleted in Cr [58]. According to Murakami et al. [59], a thin SiO_2_ layer was formed on Nb-66.7Si alloys with 10 at.%, 20 at.%, 33.3 at.% Cr additions after oxidation in flowing air at 750 °C. Al additions may not have a beneficial effect on the oxidation of NbSi_2_ at low temperature. The Nb-56Si-11Al alloy exhibited scale spallation when it was exposed to dry air at 750 °C [56]. The alloys Nb-56Si-11Al-3Cr (Cr-doped alloy) and Nb-48Si-19Al-29Cr (Cr-rich alloy) showed very good oxidation resistance at low temperature but the Cr doped alloy had very good oxidation resistance in the range of 500 °C to 1400 °C, and the Cr-rich alloy had very poor oxidation resistance at high temperatures [56].

Based on the microstructure observed in the scale/substrate interface, it is suggested that the (Cr,Ti,Nb)(Si,Al)_2_ compound presented higher Al and Si activities that made possible the formation of an outer SiO_2_ +Al_2_O_3_ scale, and an inner layer of Al_2_O_3_.

The EDS analysis of the oxidation products of the (Nb,Cr,Ti)_6_Si_5_ phase was limited owing to the very thin scale that formed on top of this phase. Images of the scale surface suggested that the oxidation products were complex rutile type oxides, SiO_2_ and some Al_2_O_3_. EDS analyses of the (Nb,Ti)(Si,Al)_2_ at the substrate/scale interface did not reveal elemental depletion, especially of Al, which was actually slightly enriched at the substrate/scale interface. Murakami et al. [59] observed a similar behaviour in the alloy Nb-47Si-20Al with the Nb_3_Si_5_Al_2_ phase as the matrix. The (Cr,Ti,Nb)(Si,Al)_2_ compound presented some Al depletion of about 50% less of the initial Al content. There is no data to compare with the (Nb,Cr,Ti)_6_Si_5_ phase.

The α-Al_2_O_3_ is not expected to form at 800 °C. However, the GXRD indicated its presence. The EDS analyses of the scale showed that there were two microstructures in the areas that were Al and O rich, one consisting of spherical clusters of angular particles which were mostly located in the Al rich areas of the alloy, and ridge networks that spread over the Al-rich areas of the (Nb,Ti)(Si,Al)_2_ phase. According to Brumm and Grabke [43], the ridge network microstructure is related to the transformation of θ-Al_2_O_3_ to α-Al_2_O_3_. If this transformation had occurred, the oxidation rate should have decreased. Instead it increased, which suggests that another contribution to the formation of less protective oxides influenced the slight increase of the oxidation rate. Thus, the above could suggest that Cr promoted a faster stabilization of α-Al_2_O_3_ at the scale/substrate interface, while at the scale/gas interface θ-Al_2_O_3_ whiskers were formed. Cr promotes the transformation of θ-Al_2_O_3_ to α-Al_2_O_3_ [43]. The oxide surface formed over some Al-rich areas in the alloy presented a network-like structure Al_2_O_3_ that extended over the scale of (Nb,Ti)(Si,Al)_2_, which suggests a lateral growth that had resulted from the transformation of θ-Al_2_O_3_ to α-Al_2_O_3_.

The oxidation of the alloy OHC5 was different from those of Nb-Si-Al based alloys reported in the literature. Although the Nb-Al-Si-Cr alloys studied by Murakami et al. [59] did not suffer from pest oxidation at 750 °C and followed parabolic oxidation, they did not form Al_2_O_3_ at low temperature, instead mixed oxides of all the components were formed. This would suggest that the presence of Ti in the alloy OHC5 was beneficial for the establishment of an Al_2_O_3_ oxide scale on top of the (Nb,Ti)(Si,Al)_2_ and (Cr,Ti,Nb)(Si,Al)_2_ phases, and that the rutile type oxides were not detrimental at 800 °C.

**OHC1-1200 °C**: The alloy showed para-linear oxidation kinetics (Table 1). According to the EDS and GXRD data, the scale was composed of the Cr_2_O_3_, SiO_2_ and TiO_2_ oxides. The Cr_2_O_3_ was the predominant oxide in the scale. The composition of this oxide was affected by the composition of the (TM)_6_Si_5_ phase and its different Ti, Fe and Cr contents (Figure 2). The Fe_2_O_3_ and Cr_2_O_3_ oxides with rhombohedral crystal structure show a continuous series of solid solutions in the Fe-Cr-O ternary system. The Fe_2_O_3_, Cr_2_O_3_ and Ti_2_O_3_ are isostructural, thus it is not surprising to find different ranges of solubility according to the availability of Fe, Cr and Ti in the (TM)_6_Si_5_. The oxidation of the complex silicide (Ti,Fe,Cr)_7_Si_6_ was reported by Portebois et al. [60]. Its oxidation products were similar to those formed on the (TM)_6_Si_5_ in OHC1 at 1200 °C except for the formation of Cr_2_TiO_5_ which seems not to be in equilibrium with Cr_2_O_3_ below 1660 °C [61].

According to Kosftad [62], during the growth of Cr_2_O_3_ internal strains arise in the scale as a result of oxygen and Cr transport through the scale with the Cr diffusion being much faster than that of the O. The Cr_2_O_3_ layer presented a granular morphology. It is likely that grain boundaries allowed the transport of oxygen further in the alloy to oxidize the FeSi_2_Ti and form SiO_2_ and some TiO_2_ at this temperature. This would explain why the SiO_2_ was mainly found below the Cr_2_O_3_. Some areas of the scale were still in contact with the Cr_2_O_3_ layer. The protectiveness of the scale formed on the alloy OHC1 would rely on the establishment of a more continuous SiO_2_ layer underneath the Cr_2_O_3_ that could act as a barrier for further metal and oxygen transport. 

The thickness of the scale was in the range 10 to 30 μm. The Cr_2_O_3_ was mostly found in the outermost layer, and the SiO_2_ in the inner part of the scale. The distribution of these oxides in the scale was irregular and was the reason for the variation in thickness. The scale was adherent, but could not be considered as protective. Cr_2_O_3_ in the scale has been linked with para-linear oxidation at high temperatures [50]. 

The insert number 1 in the Figure 18 shows coarse and fine grains of Cr_2_O_3_ in the top of the scale where the oxygen partial pressure was higher than at the scale/substrate interface. The para-linear behaviour is attributed to the fact that Cr_2_O_3_ can be further oxidised at high oxygen pressures and high temperatures to form CrO_3_, which is volatile at 1200 °C. It is likely that the mixture of coarse and fine grained Cr_2_O_3_ in the scale was the result of the reaction Cr_2_O_3_ (s) + 3/2 O_2_ (g) = 2 CrO_3_ (g), which could be responsible for the change in the oxidation of this alloy from parabolic to linear after 40 h at 1200 °C. According to Kofstad [41], the oxidation of Cr_2_O_3_ into CrO_3_ is enhanced as the thickness of Cr_2_O_3_ increases. The EDS analyses performed on Cr_2_O_3_ at different distances from the scale/substrate interface showed some Ti and Fe in solution in this oxide.

The insert number 2 in Figure 18 shows an oxide with a glassy-like appearance. Qualitative EDS showed that this was SiO_2_. These areas were mostly observed on top of the FeSi_2_Ti phase in the underlying microstructure. As was the case for the oxidation of this phase at 800 °C, TiO_2_ and SiO_2_ were its oxidation products. The EDS showed some Ti dissolved in the SiO_2_. The GXRD showed peaks that corresponded to TiO_2_. Becker et al. [63] suggested that the solubility of TiO_2_ in SiO_2_ is increased with temperature. This would be the reason why it was possible to find TiO_2_ dissolved in SiO_2_ instead of coarse particles of TiO_2_ dispersed in a SiO_2_ network. Despite the high Fe content of the FeSi_2_Ti phase, no Fe oxides were detected. This was attributed to the preferential oxidation of Si and Ti, and is in agreement with Tsirlin et al. [64]. One of the possible reasons for this behaviour is that the low Fe solubility in TiO_2_ allowed a mixture of TiO_2_ and SiO_2_ to be stabilised at 1200 °C. Indeed, according to Wittke [47] the solid solubility of Fe in TiO_2_ is about 1 at.% in the range of 800 °C to 1200 °C.

Oxide melting could be responsible for the network-like oxide microstructure observed in the insert number 3 in Figure 18. This feature was observed on the oxide formed on top of the (Fe,Cr,Ti)Si phase. It is suggested that this melting could be the result of the eutectic reaction L → FeO + Cr_2_O_3_ + SiO_2_ at 1155 °C reported by Kainarskii and Degtyareva [65]. EDS analyses from this area confirmed the presence of Si, Cr and some Fe and thus it is possible that some FeO formed. Its volume fraction could have been low to be detected by GXRD but enough to react with the SiO_2_ and Cr_2_O_3_ that were the dominant oxides.

In the cross sections of the substrate/scale interface voids were observed in the (Fe,Cr,Ti)Si phase and some Cr depletion in the substrate underneath the scale. The solid solutions formed by Fe_2_O_3_-Cr_2_O_3_ are converted at high temperatures to the ternary FeO-Fe_2_O_3_-Cr_2_O_3_ by the dissociation of Fe_2_O_3_ [65]. According to the Fe-Cr-O system, at 1200 °C the FeO dissolves Cr before some spinels are stabilized. Thus, if FeO was formed this could suggest that this phase could transport some Cr towards the oxide surface. There was some Cr in the SiO_2_ that formed on top of the (Fe,Cr,Ti)Si phase, which would suggest that there was some Cr transport from the substrate/scale interface towards the oxide/scale surface through the SiO_2_ network. The diffusion zone beneath the scale was 30–40 μm thick. 

It is likely that the oxidation of this alloy in the first 40 hours at 1200 °C involved the formation of Cr_2_O_3_ layer along with SiO_2_ layer underneath and some evaporation of CrO_3_. When the Cr_2_O_3_ reached a certain thickness the CrO_3_ evaporation became more important leading to a change in oxidation from parabolic to linear. There should have also been some contribution to the oxidation kinetics from the other minor oxides that were formed in the scale at this temperature. The overall oxidation could be considered as para-linear even though n = 0.68 (Table 1). According to Kosftad [62], para-linear oxidation occurs when a compact and protective scale forms at the scale/gas interface and becomes non-protective owing to the formation of pores caused by oxide evaporation. Then the oxidation kinetics changes from parabolic to linear. 

**OHC5-1200 °C**: The EDS and GXRD data indicated that the scale was composed of α-Al_2_O_3_, see Figure 20b, the analysis for number (1) in Figure 21 and the aluminium and oxygen X-ray maps in Figure 22. All the phases in the alloy must had contributed to α-Al_2_O_3_ formation at 1200 °C including the (Nb,Cr,Ti)_6_Si_5_ that had very low Al solubility. Figure 7 would suggest that initially some transient oxides might have had also formed.

As it is often observed in alumina scales, the Al_2_O_3_ scale formed on the alloy OHC5 had uneven thickness, oxide lumps and lace-like ridges. The growth of Al_2_O_3_ ridges is the result of the transformation of transient alumina(s) to α-Al_2_O_3_. According to Prescott et al. [66], the transformation of transient alumina to α-Al_2_O_3_ starts and grows laterally until grain boundaries meet. The last areas to convert to α-Al_2_O_3_ are the grain boundaries where ridges form as a result of the outward diffusion of Al ions thickening the oxide formed at the grain boundaries. On the oxide surface there were particles with the same composition but different morphologies, which would suggest that some deformation that resulted from the build-up of compressive stress from the oxide growth had affected the supply of Al and O for the continuous growth of Al_2_O_3_. Grain orientation and the composition of the oxidised phase may have also contributed to the different morphology presented by the Al_2_O_3_ particles. The cross sections showed that the scale was continuous and adherent and presented the classical morphology of a α-Al_2_O_3_ scale with coarsened grains at the substrate/scale interface.

### 5.3. Comparison with Alumina Scale Forming Nb-Ti-Si-Al-Hf Alloys and Nb-Silicide Based Alloys

**Figure 23:**
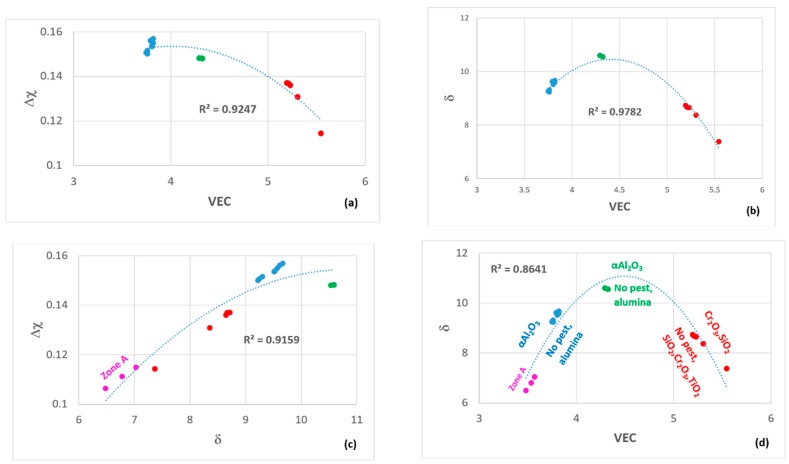
(**a**) Δχ versus VEC and (**b**) δ versus VEC maps without data for Zone A of the alloy MG7, (**c**) Δχ versus δ and (**d**) δ versus VEC maps with data for Zone A of the alloy MG7. Types of scale formed at 800 and 1200 °C are shown in (**d**). Colours: OHC1 (red), OHC5 (green), Zone A MG7 [2] (purple), MG5, MG6, MG7 [3] (blue).

The alloys OHC1 and OHC5 are shown in the maps of the parameters VEC, Δχ and δ in Figure 23 where the Nb-Ti-Si-Al-Hf alloys studied in [2,3] are included. The data for the Zone A in the alloy MG7, where a “layered” microstructure was observed, is included in the Figure 23c,d. The correlations are remarkably good in the Δχ versus VEC and δ versus VEC maps respectively in the Figure 23a,b. When the data for Zone A is included, the correlations are also good, particularly in the Δχ versus δ map in Figure 23c. The δ versus VEC map in Figure 23d indicates the scales formed at 800 °C and 1200 °C and the non-pesting behaviour at 800 °C. 

The VEC, Δχ and δ values of the alloys OHC1 and OHC5 are compared with those of Nb-silicide based alloys [67] and the Nb-Ti-Si-Al-Hf alloys MG5, MG6 and MG7 [2,3] in Table 2. The two alloys of this study had Δχ and δ within the range of Nb-silicide based alloys, as was the case for the MG series of alloys, and their VEC values were lower and higher than the Nb-silicide based alloys. Furthermore, the VEC values of the non pesting and alumina forming alloy OHC5 were lower than those of Nb-silicide based alloys, like the VEC values of the non pesting and alumina forming Nb-Ti-Si-Al-Hf alloys.

**Figure 24:**
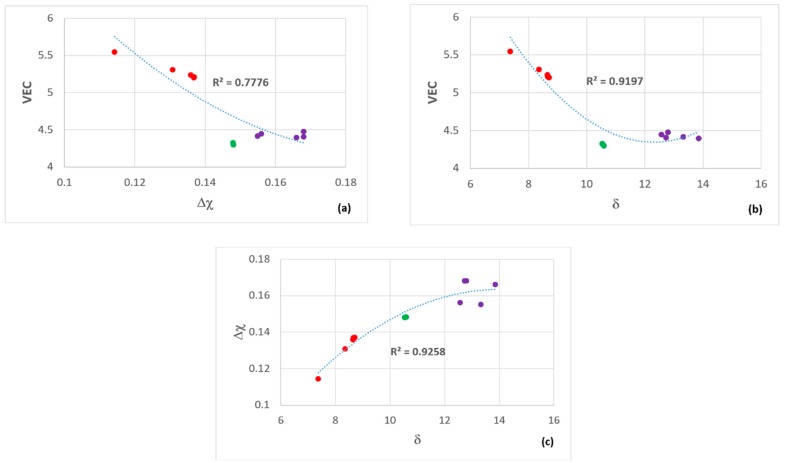
(**a**) VEC versus Δχ, (**b**) VEC versus δ and (**c**) Δχ versus δ maps of the alloys OHC1 (red), OHC5 (green) and non-pesting and oxidation resistant B containing Nb-Silicide based (purple) alloys [67].

Figure 24 shows maps of the parameters VEC, Δχ and δ where the alloys OHC1 and OHC5 correlate well with those of non-pesting and oxidation resistant B containing Nb-silicide based alloys. It should be noted that the alloys MG5, MG6 and MG7 also exhibited a good correlation with B containing Nb-silicide based alloys but only in the VEC versus δ map (see Figure 12c in [3]) and that the trend in better correlation (increase in R^2^ value) is the same as in the Figure 23.

Pathways for alumina and/or silica forming BC alloys for Nb-silicide based alloys could be selected using Figure 23 and Figure 24 and Table 2 and the data in [3]. Note that the alumina scale forming alloys MG7 [2] and OHC5 formed “layered” structures in their cast buttons.

### 5.4. Suggestions for future work

The research reported in this paper and in [2,3] presented a vision about coating alloys for BC for Nb-silicide based alloys and together with [2,3] drew attention to matters that require better understanding. Si-Fe-Cr-Ti-Nb silicide coating alloys with/out Al addition might be worth further investigation for specific families of Nb-silicide based alloys, perhaps those with C14 Laves phases in their microstructures. 

The isothermal oxidation behaviour of the alloys OHC1 and OHC5 is not enough to “guarantee” their suitability for application in coatings. The evaluation of oxidation resistance must also consider the ability of the scales formed on the alloys to resist the thermally induced stresses associated with cyclic behaviour. Future research should evaluate whether the alloys OHC1 and OHC5 have good oxidation under cyclic conditions.

A substrate alloy with inherent oxidation resistance and a balance of mechanical properties should be selected, as well as a coating process to evaluate the oxidation of the substrate/coating system under cyclic conditions. This would allow one to get an understanding of the mechanism(s) of the interaction between each coating alloy and the chosen substrate, and thus to appraise the stability of the substrate/coating interface and the contamination of the substrate by interstitials. It is hoped that the work presented in this paper will inspire new research on the development of coatings for Nb-silicide based alloys.

## 6. Summary and Conclusions

The microstructures and isothermal oxidation of the silicide based alloys 46Si-23Fe-15Cr-15Ti-1Nb (OHC1) and 60Si-25Nb-5Al-5Cr-5Ti (OHC5) were studied. The cast microstructures consisted of the (TM)_6_Si_5_, FeSi_2_Ti and (Fe,Cr)Si (OHC1), and the (Nb,Ti)(Si,Al)_2_, (Nb,Cr,Ti)_6_Si_5_, (Cr,Ti,Nb)(Si,Al)_2_ (Si)_ss_ and (Al)_ss_ (OHC5) phases. The same compounds were in equilibrium in OHC1 at 1200 °C. The (Nb,Ti)(Si,Al)_2_ and (Nb,Cr,Ti)_6_Si_5_ compounds were in equilibrium in OHC5 at 1400 °C. In OHC1 the (TM)_6_Si_5_ was the primary phase in which the Fe, Cr and Ti partitioned with k_o_^Fe^ = 0.522, k_o_^Cr^ = 1.482 and k_o_^Ti^ = 1.267, respectively. The FeSi and FeSi_2_Ti silicides formed a binary eutectic. In OHC5 the (Nb,Ti)(Si,Al)_2_ was the primary phase.

At 800 °C both alloys did not pest. The alloy OHC1 followed parabolic oxidation kinetics during the first 10 h, followed by linear kinetics and gained 1.42 mg/cm^2^ after 100 h. Its scale was composed of SiO_2_, TiO_2_ and (Cr,Fe)_2_O_3_ oxides. The FeSi_2_Ti formed SiO_2_ and TiO_2_, the (Fe,Cr)Si formed SiO_2_ with a thin layer of (Fe,Cr)_2_O_3_ on top and the (TM)_6_Si_5_ formed SiO_2_ and TiO_2_ over the centre of its dendrites and SiO_2_, TiO_2_ and (Cr,Fe)_2_O_3_ over its Fe-rich edges. The alloy OHC5 followed parabolic oxidation kinetics, gained 0.22 mg/cm^2^ after 100 h and formed a very thin and adherent scale composed of Al_2_O_3_, SiO_2_ and (Ti_(1−x−y)_,Cr_x_,Nb_y_)O_2_ oxides. The scaled formed on (Cr,Ti,Nb)(Si,Al)_2_ consisted of an outer layer that was composed of SiO_2_ and Al_2_O_3_ oxides and an inner layer of Al_2_O_3_. The scale formed on the (Nb,Cr,Ti)_6_Si_5_ was thin, consisted of (Ti_(1−x−y)_,Cr_x_,Nb_y_)O_2_ and SiO_2_ (main oxides) and some Al_2_O_3_ near the edges of the compound. In the (Nb,Ti)(Si,Al)_2_ compound the critical Al concentration for the formation of a continuous and adherent Al_2_O_3_ scale was 3 at.%. Below this Al concentration an internal oxidation zone formed below the scale/substrate interface.

At 1200 °C the alloy OHC1 followed para-linear oxidation kinetics and gained 1.60 mg/cm^2^ after 100 h. The scale was composed of a SiO_2_ inner layer and outer layers of Cr_2_O_3_ and TiO_2_. There was internal oxidation and CrO_3_ evaporation. Most likely the eutectic reaction L → FeO + Cr_2_O_3_ + SiO_2_ had occurred in the scale. The weight gain of the alloy OHC5 was 0.85 mg/cm^2^ after 100 h. The scale was composed of α-Al_2_O_3_.

Both alloys exhibited good correlations with alumina forming Nb-Ti-Si-Al-Hf alloys and with B containing Nb-silicide based alloys in maps of the parameters δ (related to atomic size), Δχ (related to electronegativity) and number of valence electrons per atom filled into the valence band (VEC). 

The alloy OHC1 could not be used in a coating system above 1100 °C because, even though it had good oxidation resistance at 800 °C and 1200 °C, and formed a SiO_2_ sublayer, it would suffer from insipient melting at about 1300 °C, melting in the scale at about 1155 °C and the evaporation of CrO_3_. Future research could address the effects of alloying on microstructure stability and oxidation resistance of variants of the alloy OHC1. Compared with the alloy OHC1, the alloy OHC5 could be a candidate coating alloy worthy of further consideration regarding alloy development for a BC application owing to its ability to form alumina scales even at such a low Al concentration in the alloy. 

## Figures and Tables

**Table 1:** Total weight gain, n values and oxidation rate constants of the alloys OHC1 and OHC5 after isothermal oxidation at 800 °C and 1200 °C.

Alloy and Temperature	n	k_l_ (g·cm^−2^·s^−1^)	k_p_ (g^2^·cm^−4^·s^−1^)	Weight Gain (mg/cm^2^)
OHC1-800 °C	0.89	3.9 × 10^−9^ > 10 h	5.47 × 10^−13^ (0–10 h)	1.42
OHC1-1200 °C	0.68	3.74 × 10^−9^ > 40 h	4.13 × 10^−11^ (0–40 h)	1.60
OHC5-800 °C	0.54	-	3.4 × 10^−13^ (0–1.3 h) 3.8 × 10^−14^ (1.3–24 h) 1.5 × 10^−13^ (>24 h)	0.22
OHC5-1200 °C	-	4.4 × 10^−8^ (0–4.5 h) 2.1 × 10^−8^ (17–21.5 h)	1.41 × 10^−12^ (>21.5 h)	0.85

**Table 2:** Values of the parameters Δχ, δ and VEC for Nb-silicide based alloys and the alloys OHC1, OHC5, MG5, MG6 and MG7.

Alloy	VEC	Δχ	δ	Ref
OHC1	5.23 *	0.135 *	8.57 *	This work
OHC5	4.3 *	0.148 *	10.54 *	This work
MG5	3.84 ^+^	0.157 ^+^	9.75 ^+^	[3]
MG6	3.75 ^+^	0.15 ^+^	9.25 ^+^	[3]
MG7	3.77 ^+^	0.157 ^+^	9.7 ^+^	[2]
MG7 zone A	3.53 ^+^	0.11 ^+^	6.77 ^+^	[2]
Nb-silicide based alloys	4.37 to 4.9	0.12 to 0.237	8.1 to 14.3	[67]

* for nominal composition, ^+^ average value.
